# The MicroRNA *mir-71* Inhibits Calcium Signaling by Targeting the TIR-1/Sarm1 Adaptor Protein to Control Stochastic L/R Neuronal Asymmetry in *C. elegans*

**DOI:** 10.1371/journal.pgen.1002864

**Published:** 2012-08-02

**Authors:** Yi-Wen Hsieh, Chieh Chang, Chiou-Fen Chuang

**Affiliations:** Division of Developmental Biology, Cincinnati Children's Hospital Medical Center Research Foundation, Cincinnati, Ohio, United States of America; University of California San Diego, United States of America

## Abstract

The *Caenorhabditis elegans* left and right AWC olfactory neurons communicate to establish stochastic asymmetric identities, AWC^ON^ and AWC^OFF^, by inhibiting a calcium-mediated signaling pathway in the future AWC^ON^ cell. NSY-4/claudin-like protein and NSY-5/innexin gap junction protein are the two parallel signals that antagonize the calcium signaling pathway to induce the AWC^ON^ fate. However, it is not known how the calcium signaling pathway is downregulated by *nsy-4* and *nsy-5* in the AWC^ON^ cell. Here we identify a microRNA, *mir-71*, that represses the TIR-1/Sarm1 adaptor protein in the calcium signaling pathway to promote the AWC^ON^ identity. Similar to *tir-1* loss-of-function mutants, overexpression of *mir-71* generates two AWC^ON^ neurons. *tir-1* expression is downregulated through its 3′ UTR in AWC^ON^, in which *mir-71* is expressed at a higher level than in AWC^OFF^. In addition, *mir-71* is sufficient to inhibit *tir-1* expression in AWC through the *mir-71* complementary site in the *tir-1* 3′ UTR. Our genetic studies suggest that *mir-71* acts downstream of *nsy-4* and *nsy-5* to promote the AWC^ON^ identity in a cell autonomous manner. Furthermore, the stability of mature *mir-71* is dependent on *nsy-4* and *nsy-5*. Together, these results provide insight into the mechanism by which *nsy-4* and *nsy-5* inhibit calcium signaling to establish stochastic asymmetric AWC differentiation.

## Author Summary

Cell identity determination requires a competition between the induction of cell type–specific genes and the suppression of genes that promote an alternative cell type. In the nematode *C. elegans*, a specific sensory neuron pair communicates to establish stochastic asymmetric identities by inhibiting a calcium signaling pathway in the neuron that becomes an induced identity. However, it is not understood how cell–cell communication inhibits the calcium signaling pathway in the induced neuronal identity. In this study, we identify a microRNA that represses the expression of a key molecule in the calcium signaling pathway to promote the induced neuronal identity. Overexpression of the microRNA causes both neurons of the pair to become the induced identity, similar to the mutants that lose function in the calcium signaling pathway. In addition, the stability of the mature microRNA is dependent on a claudin-like protein and a gap junction protein, the two parallel signals that mediate communication of the neuron pair to promote the induced neuronal identity. Our results provide insight into the mechanism by which cell–cell communication inhibits calcium signaling to establish stochastic asymmetric neuronal differentiation.

## Introduction

Cell fate determination during development requires both the induction of cell type specific genes and the suppression of genes that promote an alternative cell fate [1]–[4]. For example, both inductive signaling, mediated by an EGFR-Ras-MAPK pathway, and lateral inhibition, mediated by LIN-12/Notch activity and microRNA (miRNA), are required for six multipotential vulval precursor cells to adopt an invariant pattern of fates in *C. elegans* [5]. Notch signaling-mediated lateral inhibition also plays a crucial role in the neuronal/glial lineage decisions of neural stem cells; as well as the B/T, alphabeta/gammadelta, and CD4/CD8 lineage choices during lymphocyte development [6], [7]. In the *Drosophila* eye, the kinase Warts and PH-domain containing Melted repress each other's transcription in a bistable feedback loop to regulate the two alternative R8 photoreceptor subtypes expressing Rhodopsin Rh5 or Rh6 [2]. In the *C. elegans* sensory system, two sets of transcription factors and miRNAs reciprocally repress each other to achieve and stabilize one of the two mutually exclusive ASEL and ASER taste neuronal fates [8]–[10]. Notch signaling acts upstream of the miRNA-controlled bistable feedback loop to regulate ASE asymmetry through a lineage-based mechanism in early embryos [11].

The *C. elegans* left and right sides of Amphid Wing Cell C (AWC) olfactory neurons specify asymmetric subtypes through a novel mechanism independent of the Notch pathway in late embryogenesis [12]. Like ASE neurons, the two AWC neurons are morphologically symmetrical but take on asymmetric fates, such that the AWC^ON^ neuron expresses the chemoreceptor gene *str-2* and the contralateral AWC^OFF^ neuron does not [12]–[14]. Asymmetric differentiation of AWC neurons allows the worm to discriminate between different odors [15]. In contrast to reproducible ASE asymmetry, AWC asymmetry is stochastic: 50% of animals express *str-2* on the left and the other 50% express it on the right. Ablation of either AWC neuron causes the remaining AWC neuron to become AWC^OFF^, suggesting that AWC^OFF^ is the default state and the induction of AWC^ON^ requires an interaction or competition between the AWC neurons [12]. The axons of the two AWC neurons form chemical synapses with each other; AWC asymmetry is established near the time of AWC synapse formation [16], [17]. In addition, axon guidance mutants are defective in inducing the AWC^ON^ state. These results suggest that the synapses could mediate the AWC interaction for asymmetry [12].

*nsy-4*, encoding a claudin-like tight junction protein, and *nsy-5*, encoding an innexin gap junction protein, act in parallel to downregulate the calcium-mediated UNC-43 (CaMKII)/TIR-1 (Sarm1)/NSY-1 (MAPKKK) signaling pathway in the future AWC^ON^ cell [18], [19]. Both AWCs and non-AWC neurons in the NSY-5 gap junction dependent cell network communicate to participate in signaling that coordinates left-right AWC asymmetry. In addition, non-AWC neurons in the NSY-5 gap junction network are required for the feedback signal that ensures precise AWC asymmetry [18]. Once AWC asymmetry is established in late embryogenesis, both the AWC^ON^ and AWC^OFF^ identities are maintained by cGMP signaling, dauer pheromone signaling, and transcriptional repressors [12], [20], [21]. *unc-43*(CaMKII), *tir-1* (Sarm1), and *nsy-1* (MAPKKK) are also implicated in the maintenance of AWC asymmetry in the first larval (L1) stage [22]. Although multiple genes were identified to be involved in the establishment and the maintenance of AWC asymmetry (for a review, see [23]), it is still unknown how the calcium-regulated signaling pathway is inhibited by *nsy-4* and *nsy-5* in the AWC^ON^ cell.

The TIR-1/Sarm1 adaptor protein assembles a calcium-signaling complex, UNC-43 (CaMKII)/TIR-1/NSY-1 (ASK1 MAPKKK), at AWC synapses to regulate the default AWC^OFF^ identity [16], thus downregulation of *tir-1* expression may represent an efficient mechanism to inhibit calcium signaling in the cell becoming AWC^ON^. In support of this idea, a prior large scale examination of potential miRNA targets indicated that *tir-1* and *unc-43* may be downregulated by this class of RNAs [24]. Here, we analyze the function of the miRNA *mir-71* in stochastic AWC asymmetry by characterizing its role in downregulation of the calcium signaling pathway in the AWC^ON^ cell. We show that *mir-71* acts downstream of *nsy-4*/claudin and *nsy-5*/innexin to promote AWC^ON^ in a cell autonomous manner through inhibiting *tir-1* expression, in parallel with other processes. We also show that *nsy-4* and *nsy-5* are required for the stability of mature *mir-71*. Our results suggest a mechanism for genetic control of AWC asymmetry by *nsy-4* and *nsy-5* through *mir-71*-mediated downregulation of calcium signaling.

## Results

### Identification of miRNAs with predicted target genes in the AWC calcium signaling pathway

The calcium-regulated UNC-43 (CaMKII)/TIR-1 (Sarm1)/NSY-1 (ASK1 MAPKKK) signaling pathway suppresses expression of the AWC^ON^ gene *str-2* in the default AWC^OFF^ cell [12], [16], [25], [26]. To establish AWC asymmetry, the calcium-mediated signaling pathway is suppressed in the future AWC^ON^ cell. miRNAs are small non-coding RNAs that are robust in mediating post-transcriptional and/or translational downregulation of target genes [27]. In *C. elegans*, miRNAs are processed from premature form into mature form by *alg-1/alg-2* (encoding the Argonaute proteins) and *dcr-1* (encoding the ribonuclease III enzyme Dicer) [28]. Gene expression profiling revealed increased levels of *unc-43* and *tir-1* in *dcr-1* mutants [24], suggesting that *unc-43* and *tir-1* may be downregulated by miRNAs. Thus, we hypothesized that miRNAs may play a role in downregulation of the UNC-43/TIR-1/NSY-1 signaling pathway in the cell becoming AWC^ON^.

To test this hypothesis, we took a computational approach to identify miRNAs predicted to target the 3′ UTRs of known genes, including *unc-2*, *unc-36*, *egl-19*, *unc-43*, *tir-1*, *nsy-1*, and *sek-1*, in the AWC calcium signaling pathway. Only the miRNAs that fit the following criteria were selected for further analysis: 1) At least 6 nucleotides in the seed region (position 1–7 or 2–8 at the 5′ end) of a miRNA is perfectly matched to the target 3′ UTR; 2) The seed match between a miRNA and its target 3′ UTR is conserved between *C. elegans* and a closely related nematode species *C. briggsae*, since evolutionary conservation between *C. elegans* and *C. briggsae* genomes is useful in identifying functionally relevant DNA sequences such as regulatory regions [29], [30]; and 3) A miRNA is predicted by both MicroCosm Targets (formerly miRBase Targets; http://www.ebi.ac.uk/enright-srv/microcosm/htdocs/targets/v5/) [31]–[33] and TargetScan (http://www.targetscan.org/worm_12/) [34]. Based on these criteria, we identified six potential miRNAs (*mir-71*, *mir-72*, *mir-74*, *mir-228*, *mir-248*, *mir-255*) predicted to target *unc-2*, *unc-43*, *tir-1*, *nsy-1*, and *sek-1* (Figure S1A). A subset of these identified miRNA-target pairs were also predicted by other miRNA target prediction programs, including PicTar (http://pictar.mdc-berlin.de/) [35] and mirWIP (http://146.189.76.171/query.php) [36].

**Figure 1:**
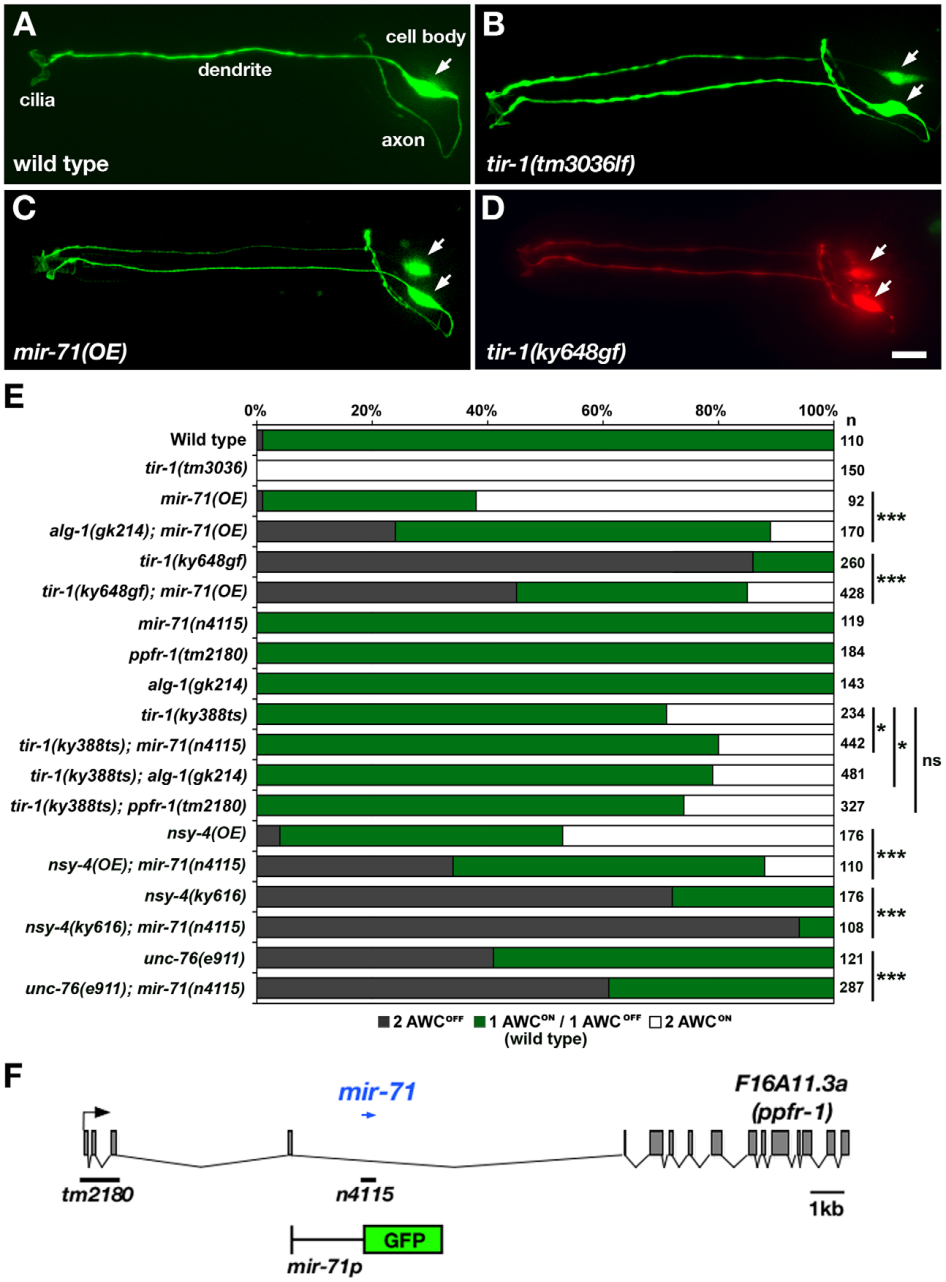
*mir-71* promotes the AWC^ON^ identity. (A–D) Expression of a stable transgene *str-2p::GFP* (AWC^ON^ marker) in wild type (A), *tir-1(tm3036)* loss-of-function (lf) mutants (B), *mir-71(OE)* animals overexpressing the transgene *odr-3p::mir-71* in AWCs (C), and *tir-1(ky648gf)* mutants (D). *tir-1(ky648gf)* mutants also carry the transgene *odr-1p::DsRed* (expressed in both AWC^ON^ and AWC^OFF^) to show that the absence of *str-2p::GFP* expression is not due to loss of AWC neurons. (E) *str-2p::GFP* expression phenotypes in wild type, single mutants, and double mutants. *nsy-4(OE)* animals overexpress the transgene *odr-3p::nsy-4* in AWCs. (F) Genetic map of *mir-71. mir-71* (blue arrow) is located in an intron of *F16A11.3a* encoding the *ppfr-1* gene. Black bars indicate the location of deletions in *ppfr-1(tm2180)* and *mir-71(n4115)* mutants. A schematic of the GFP reporter gene driven by a 2.4 kb region upstream of *mir-71* transcript is shown. Arrows, AWC cell body. Scale bar, 10 µm. Statistical analysis was performed using the *Z*-test for two proportions: *p<0.05; ***p<0.001; ns, not significant.

Since most miRNAs are not individually essential and have functional redundancy [37]–[40], loss-of-function mutations in a single miRNA may not show a defect in AWC asymmetry. To circumvent potential problems that may be posed by functional redundancy, we took an overexpression approach to determine the role of these six miRNAs in AWC asymmetry. We generated transgenic strains overexpressing individual miRNAs in both AWCs using an *odr-3* promoter, expressed strongly in AWC neuron pair and weakly in AWB neuron pair [41]. Wild-type animals have *str-2p::GFP* (AWC^ON^ marker) expression in only one of the two AWC neurons (Figure 1A and 1E). Since loss-of-function mutations in the AWC calcium signaling genes (*unc-2*, *unc-36*, *unc-43*, *tir-1*, *nsy-1*, and *sek-1*) led to *str-2p::GFP* expression in both AWC neurons (2AWC^ON^ phenotype) (Figure 1B and 1E) [12], [16], [25], [26], we proposed that overexpression of the miRNA downregulating one of these calcium signaling genes would also cause a 2AWC^ON^ phenotype. We found that *mir-71(OE)* animals overexpressing *mir-71*, predicted to target *tir-1* and *nsy-1*, had a strong 2AWC^ON^ phenotype (Figure 1C, 1E, and Figure S1B). This result suggests that *mir-71* may downregulate the expression of *tir-1* and *nsy-1* to control the AWC^ON^ fate and that *mir-71* is sufficient to promote AWC^ON^ when overexpressed. However, overexpression of the other five miRNAs individually caused a mixed weak phenotype of 2AWC^ON^ and 2AWC^OFF^ (Figure S1B). Since the activity of the *nsy-1* 3′ UTR in AWC was independent of *mir-71(OE)* (Figure S2B), we focused on the investigation of the potential role of *mir-71* in promoting AWC^ON^ through negatively regulating *tir-1* expression.

### *mir-71* antagonizes the calcium signaling pathway to promote the AWC^ON^ identity

The genetic interaction between *mir-71* and *tir-1* was characterized by double mutants (Figure 1E). *tir-1(ky648)* gain-of-function (gf) mutants had two AWC^OFF^ neurons (2AWC^OFF^ phenotype) (Figure 1D and 1E) [22]. We found the *tir-1(ky648gf)* 2AWC^OFF^ phenotype was significantly reduced in the *tir-1(ky648gf)*; *mir-71(OE)* double mutants (*p*<0.001) (Figure 1E). These results support the hypothesis that *mir-71* downregulates *tir-1* to control the AWC^ON^ fate.

To further determine the requirement of *mir-71* in AWC asymmetry, we analyzed *str-2p::GFP* expression in the *mir-71(n4115)* deletion null allele [40]. *mir-71(n4115)* mutants displayed wild-type AWC asymmetry (Figure 1E), suggesting that *mir-71* may function redundantly with other miRNAs or non-miRNA genes to regulate calcium signaling in AWC asymmetry. In addition to *mir-71*, *mir-248* was also predicted to target *tir-1* by three programs (Figure S1A). *mir-71* and *mir-248* have different predicted target sites in the *tir-1* 3′ UTR. Since *mir-248* mutants are not available, we analyzed the effect of *mir-248* overexpression on AWC asymmetry. Unlike the highly penetrant 2AWC^ON^ phenotype caused by *mir-71* overexpression, *mir-248* overexpression generated a mixed weak phenotype of 2AWC^ON^ and 2AWC^OFF^ (Figure S1B). To test whether *mir-71* and *mir-248* have a synergistic effect on AWC symmetry, we made transgenic animals overexpressing both *mir-71* and *mir-248* in AWCs. The 2AWC^ON^ phenotype was not significantly higher in *mir-71(OE); mir-248(OE)* animals than in *mir-71(OE)* (data not shown). These results suggest that *mir-71* may not act redundantly with *mir-248* to regulate *tir-1* expression in AWC asymmetry. To knockdown *mir-248* expression, we made an anti-*mir248* transgene expressing short hairpin RNA (shRNA), consisting of both sense and antisense sequences of *mir-248*, in AWC. The anti-*mir-248* transgene caused an AWC phenotype similar to *mir-248(OE)* (data not shown), suggesting that the effect of the anti*-mir-248* transgene on AWC asymmetry is not through knockdown of *mir-248* but mainly due to overexpression of sense *mir-248* in the shRNA construct.

Functional redundancy of miRNAs and other regulatory pathways has been suggested by a previous study in the *Drosophila* eye [42]. To overcome functional redundancy of *mir-71*, we crossed *mir-71(n4115)* into sensitized backgrounds including *tir-1(ky388)*, *nsy-4(ky616)*, and *unc-76(e911)* mutants. *tir-1(ky388)* is a temperature-sensitive (ts) allele that caused a 2AWC^ON^ phenotype in 29% of animals at 15°C (Figure 1E) [16]. The 2AWC^ON^ phenotype of *tir-1(ky388ts)* mutants was significantly suppressed by *mir-71(n4115)*, such that 20% of *mir-71(n4115)*; *tir-1(ky388ts)* double mutants had a 2AWC^ON^ phenotype (*p*<0.05; Figure 1E). These results further support the hypothesis that *mir-71* antagonizes the function of *tir-1* in the calcium signaling pathway to promote the AWC^ON^ fate.

*mir-71* is located within a large intron of the *F16A11.3a (ppfr-1)* gene, encoding a protein phosphatase 2A regulatory subunit (Figure 1F). It is possible that the 181 bp deletion mutation within the intron of *ppfr-1* in *mir-71(n4115)* mutants may affect *ppfr-1* activity leading to suppression of the *tir-1(ky388ts)* 2AWC^ON^ phenotype. To test this possibility, we analyzed AWC phenotypes in *ppfr-1(tm2180); tir-1(ky388ts)* double mutants. *ppfr-1(tm2180)* has a 1027 bp deletion removing the first three exons and therefore is a potential null allele (Figure 1F) [43]. The 2AWC^ON^ phenotype of *ppfr-1(tm2180); tir-1(ky388ts)* double mutants was not significantly different from that of *tir-1(ky388ts)* single mutants (Figure 1E). This result suggest that *ppfr-1* is not required for AWC asymmetry and that suppression of the *tir-1(ky388ts)* 2AWC^ON^ phenotype was most likely caused by loss of *mir-71* activity in *mir-71(n4115)* mutants.

The *nsy-4* claudin-like gene and the *unc-76* axon guidance pathway gene induce the AWC^ON^ state by inhibiting the downstream calcium-signaling pathway. Loss-of-function mutations in *nsy-4* and *unc-76* cause a partially penetrant 2AWC^OFF^ phenotype (Figure 1E) [12], [19]. *mir-71(n4115)* mutations significantly enhanced the 2AWC^OFF^ phenotype of *nsy-4(ky616)* and *unc-76(e911)* mutants (*p*<0.001). On the other hand, the 2 AWC^ON^ phenotype of *nsy-4(OE)* trasnsgenic animals overexpressing *nsy-4* in AWCs was significantly suppressed in *nsy-4(OE); mir-71(n4115)* double mutants (*p*<0.001; Figure 1E). These results are consistent with a role of *mir-71* function in promoting the AWC^ON^ fate, and suggest that *mir-71* may act in parallel with other regulatory molecules to antagonize the calcium-regulated signaling pathway to generate the AWC^ON^ identity.

### *mir-71* inhibits *tir-1* expression through its 3′ UTR

**Figure 2:**
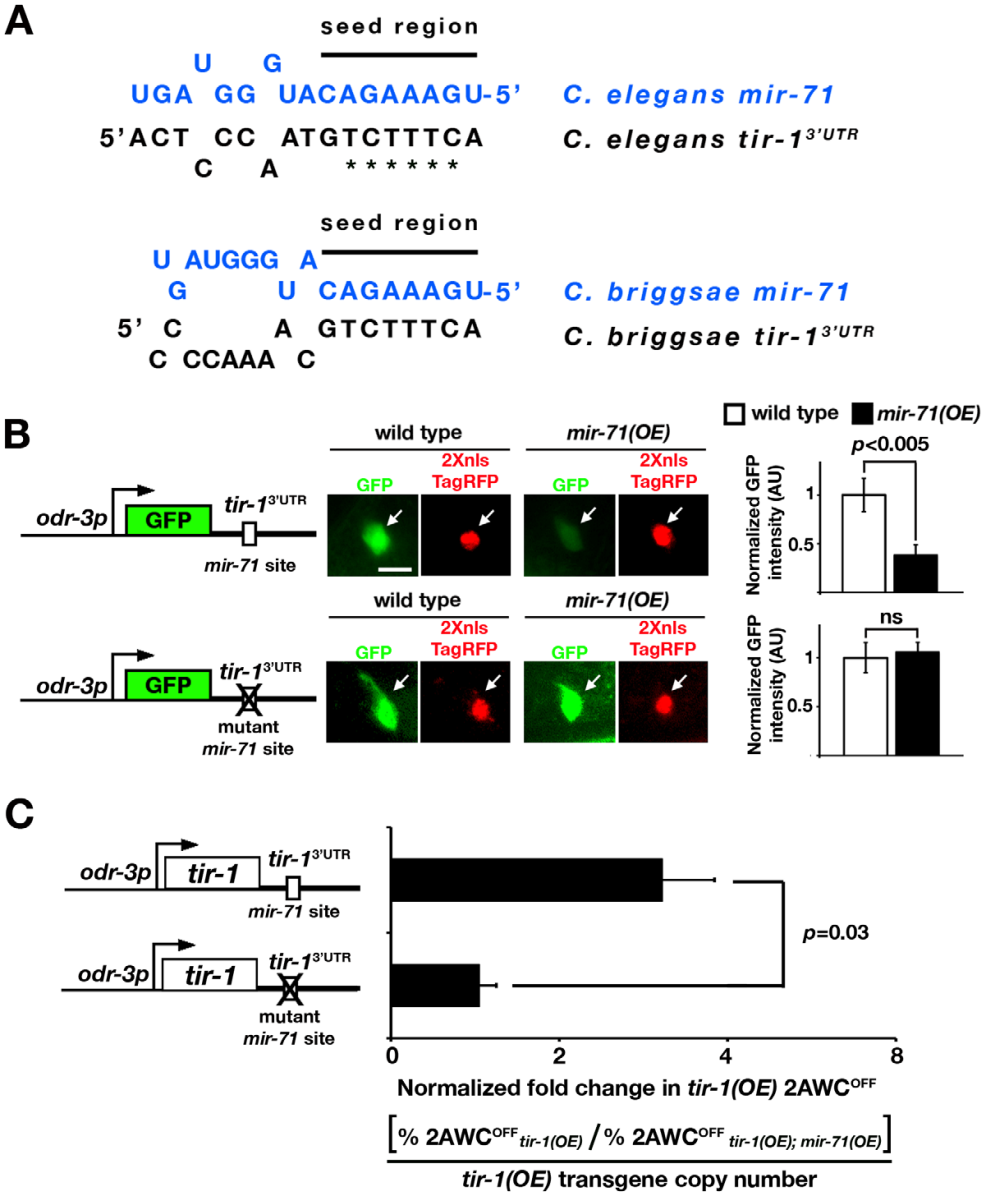
*mir-71* downregulates gene expression through the *tir-1* 3′ UTR. (A) Complementarity between the *mir-71* seed region and the *tir-1* 3′ UTR in *C. elegans* and *C. briggsae*. Asterisks denote nucleotides mutated in the predicted *mir-71* target site of the *tir-1* 3′ UTR in (B). (B) Left: GFP sensor constructs, driven by the *odr-3* promoter, with the *tir-1* 3′ UTR or the *tir-1* 3′ UTR mutated in the predicted *mir-71* target site. Middle: Images of GFP expression from GFP sensor constructs and nucleus-localized TagRFP expression from the internal control transgene *odr-3p::2Xnls-TagRFP::unc-54 3′ UTR* in the AWC cell body of wild type and *mir-71(OE)* animals. All images were taken from animals in the first larval stage. Scale bar, 5 µm. Arrows, AWC cell body. Right: The average normalized GFP intensity of each sensor construct in the AWC cell body. The GFP intensity of an individual cell was normalized to the TagRFP intensity of the same cell. For each sensor construct line, the normalized GFP intensity in wild type was set as 1 arbitrary unit (AU) and the normalized GFP intensity in *mir-71(OE)* was calibrated to that in wild type. Student's *t*-test was used for statistical analysis. n = 16–21 for each transgenic line in wild type and *mir-71(OE)* animals. Error bars, standard error of the mean. ns, not significant. (C) Left: *tir-1* overexpression constructs, driven by the *odr-3* promoter, with the *tir-1* 3′ UTR or the *tir-1* 3′ UTR mutated in the predicted *mir-71* target site. Right: Normalized fold change in *tir-1(OE)* 2AWC^OFF^ phenotype. The fold change in *tir-1(OE)* 2AWC^OFF^ phenotype was determined by dividing the 2AWC^OFF^ percentage of *tir-1(OE)* with the 2AWC^OFF^ percentage of *tir-1(OE); mir-71(OE)*, which was then normalized to the relative *tir-1(OE)* transgene copy number. Two to three independent lines were analyzed for each *tir-1* overexpression construct. Student's *t*-test was used to calculate statistical significance. Error bars represent standard error of the mean.

The predicted *mir-71* target site in the *tir-1* 3′ UTR is 96 bp downstream of the stop codon; the prediction is strongly supported by four different programs, including MicroCosm Targets, TargetScan, PicTar, and mirWIP (Figure S1A). The nucleotides at position 1–8 in the seed region of *mir-71* perfectly match the target site of the *tir-1* 3′ UTR; the seed match is conserved between *C. elegans* and *C. briggsae* (Figure 2A).

To determine whether *mir-71* acts directly through the predicted binding site in the *tir-1* 3′ UTR, we made GFP sensor constructs with the AWC *odr-3* promoter and different 3′ UTRs: wild-type *tir*-1 3′ UTR or the *tir-1* 3′ UTRmut with mutated *mir-71* target site (Figure 2B). Transgenic animals expressing each sensor construct were crossed to *mir-71(OE)* animals. The GFP intensity of each sensor construct in an individual AWC neuron was normalized to the nucleus-localized TagRFP intensity of the transgene *odr-3p::2Xnls-TagRFP::unc-54 3′ UTR* in the same cell. The *unc-54* 3′ UTR does not contain any strongly predicted *mir-71* sites. The normalized GFP intensity of each sensor construct was compared between *mir-71(OE)* animals and their siblings losing the *mir-71(OE)* transgene in the L1 stage, during which *tir-1* is functional for the maintenance of AWC asymmetry [22]. We found that *mir-71(OE)* animals, compared with wild type, had a significantly reduced normalized expression level of GFP from the *tir-1* 3′ UTR sensor construct (*p*<0.005; Figure 2B upper panels). However, the normalized expression level of GFP from the *tir-1* 3′ UTRmut was not significantly different between wild-type and *mir-71(OE)* animals (Figure 2B bottom panels). These results suggest that *mir-71* directly inhibits gene expression through the predicted target site in the *tir-1* 3′ UTR. However, we did not observe a significant difference in the GFP expression level from the *tir-1* 3′ UTR between wild-type animals and *mir-71(n4115lf)* mutants (Figure S2A). This result suggests potential functional redundancy of *mir-71* in the regulation of *tir-1* expression.

Interactions between the 5′ and 3′ UTRs have been shown to regulate translation in mammalian cells [44], bacteria [45], and RNA viruses [46]. To determine if the *tir-1* 5′ UTR plays a role in regulating the inhibitory effect of *mir-71* on the *tir-1* 3′ UTR, we included the *tir-1* 5′ UTR in the GFP sensor constructs (Figure S3). Similar to the *tir-1* 3′ UTR sensor constructs without the *tir-1* 5′ UTR (Figure 2B), the normalized expression level of GFP from the *tir-1* 5′ UTR/*tir-1* 3′ UTR sensor construct was significantly decreased in *mir-71(OE)* animals compared with wild type (*p*<0.04; Figure S3A). However, the normalized expression level of GFP from the *tir-1* 5′ UTR/*tir-1* 3′ UTRmut sensor construct was not significantly different between wild-type and *mir-71(OE)* animals (Figure S3B). These results suggest that the *tir-1* 5′ UTR does not affect *mir-71(OE)*-mediated suppression of gene expression through the *tir-1* 3′ UTR.

The *nsy-1* 3′ UTR was also predicted to contain a *mir-71* binding site by the four programs used in this study (Figure S1A), but the GFP expression level from the *nsy-1* 3′ UTR was not significantly different between wild-type and *mir-71(OE)* animals (Figure S2B). This result suggests that the predicted *mir-71* site in the *nsy-1* 3′ UTR may not be functional in AWC cells, therefore we did not further investigate the regulation of *nsy-1* expression by *mir-71*.

*tir-1(OE)* animals overexpressing *tir-1* in AWC had a 2AWC^OFF^ phenotype [16]. We used the *tir-1(OE)* 2AWC^OFF^ phenotype as readout to determine if *mir-71* acts through the *tir-1* 3′ UTR to suppress the AWC^OFF^ fate. We made *tir-1(OE)* sensor constructs by replacing GFP in the GFP sensor constructs (Figure 2B) with *tir-1* and crossed transgenic animals expressing each *tir-1(OE)* sensor construct into *mir-71(OE)* animals (Figure 2C). The fold change in *tir-1(OE)* 2AWC^OFF^ phenotype was determined by dividing the 2AWC^OFF^ percentage of *tir-1(OE)* animals with the 2AWC^OFF^ percentage of their *tir-1(OE); mir-71(OE)* siblings, which was then normalized to the relative *tir-1(OE)* transgene copy number determined by qPCR. The higher normalized fold change in *tir-1(OE)* 2AWC^OFF^ indicates more suppression of 2AWC^OFF^ phenotype by *mir-71(OE)* in *tir-1(OE); mir-71(OE)* animals. The normalized fold change in *tir-1(OE)* 2AWC^OFF^ of *tir-1* 3′ UTR was significantly higher than that of the *tir-1* 3′ UTRmut (*p* = 0.03; Figure 2C). These results suggest that *mir-71* suppresses the AWC^OFF^ fate by downregulating *tir-1* expression through its 3′ UTR.

### *mir-71* is expressed at a higher level in the AWC^ON^ cell than in the AWC^OFF^ cell

**Figure 3:**
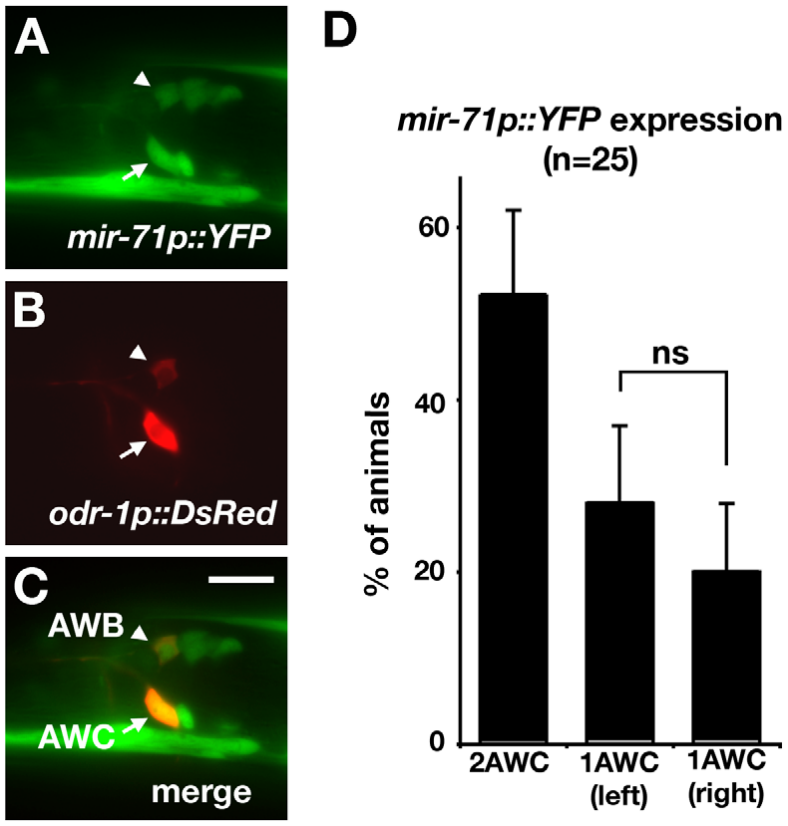
*mir-71* is expressed in AWC. (A, B) Images of a first stage larva expressing the transgenes *mir-71p::YFP* (A) and *odr-1p::DsRed*, a marker for AWB and AWC neurons (B). (C) Merged image showing co-expression of YFP and DsRed in AWC and AWB neurons. (D) Quantification of the number of AWC neurons with visible expression of the *mir-71p::YFP* reporter gene at the first larval stage. *Z*-test was used to calculate statistical significance. Error bar represents the standard error of proportion. ns, not significant. Arrowhead, AWB cell body; arrow, AWC cell body. Scale bar, 10 µm.

To determine if *mir-71* is expressed in AWC neurons, we generated transgenic animals expressing YFP under the control of a 2.4 kb promoter upstream of the *mir-71* transcript (Figure 1F). The expression of YFP was detected in several head neurons and the body wall muscle in L1 (Figure 3A), which is consistent with previously reported expression pattern of *mir-71* [47]–[49]. The *mir-71p::YFP* transgenic animals were crossed into an *odr-1p::DsRed* strain, expressing DsRed primarily in AWC and AWB neurons (Figure 3B). YFP was coexpressed with DsRed in AWC and AWB neurons (Figure 3C), suggesting that *mir-71* is expressed in these neurons. We found that 52% of animals had visible *mir-71p::YFP* in both AWC cells, 28% had visible YFP in only AWC left (AWCL), and 20% had visible YFP in only AWC right (AWCR) (Figure 3D). These results suggest that the expression of *mir-71*, when detected in one of the two AWC neurons, does not have a side bias towards AWCL or AWCR, which is consistent with stochastic choice of the AWC^ON^ fate.

**Figure 4:**
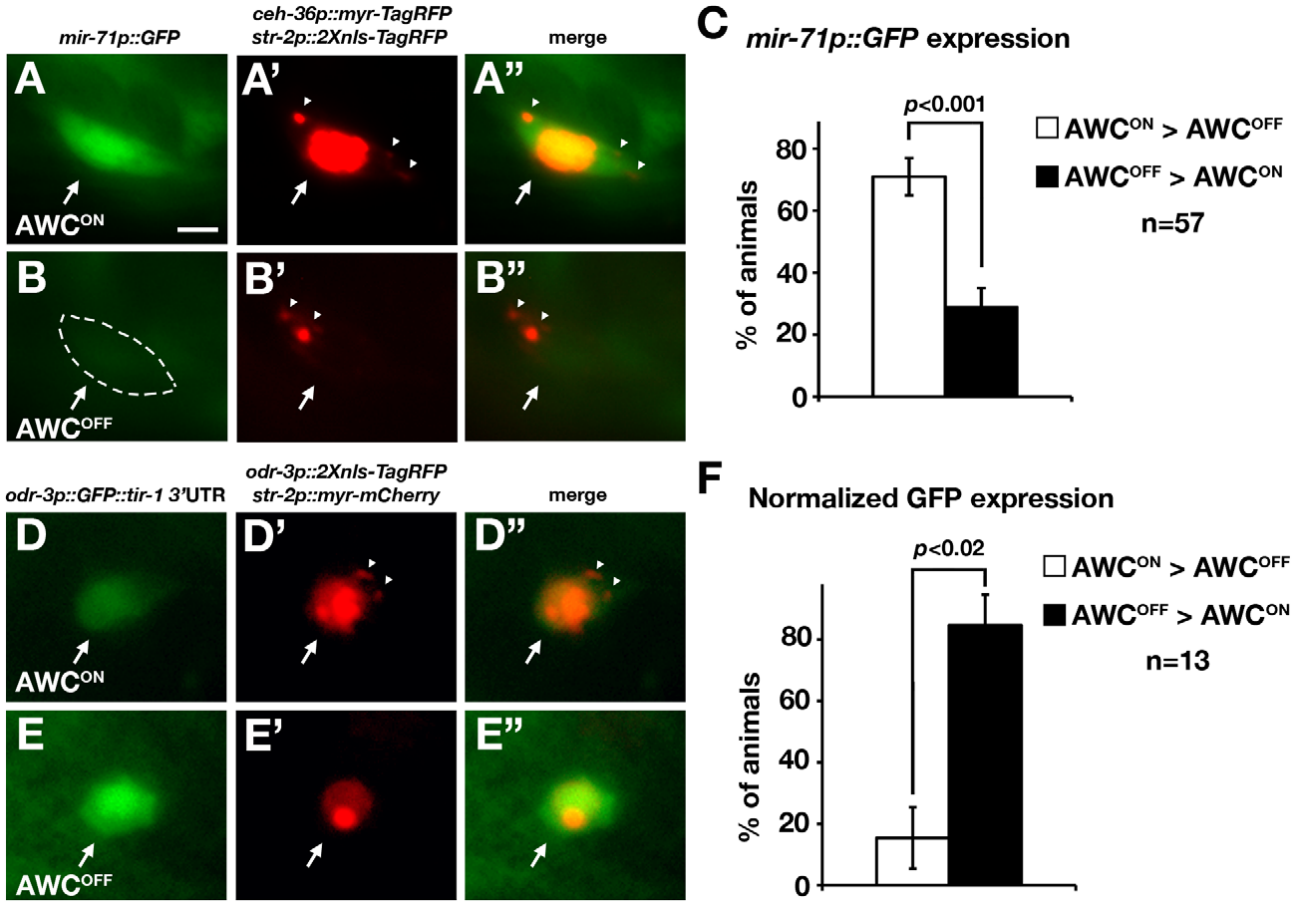
*mir-71* expression and the *tir-1* 3′ UTR are differentially regulated in AWC^ON^ and AWC^OFF^ neurons. (A, B) Images of *mir-71p::GFP*. The AWC^OFF^ cell body is outlined by dashed lines, which was done when the GFP intensity was temporarily enhanced with the Photoshop levels tool. (A′, B′) Images of *ceh-36p::myr-TagRFP* and *str-2p::2Xnls-TagRFP*. AWC^ON^ was identified as *str-2p::2Xnls-TagRFP* positive and *ceh-36p::myr-TagRFP* positive (A′). AWC^OFF^ was identified as *str-2p::2Xnls-TagRFP* negative and *ceh-36p::myr-TagRFP* positive (B′). (A″) Merge of A and A′ images from the same cell. (B″) Merge of B and B′ images from the same cell. (C) Quantification of *mir-71p::GFP* expression in AWC^ON^ and AWC^OFF^ cells. (D, E) Images of *odr-3p::GFP::tir-1 3′ UTR*. (D′, E′) Images of *odr-3p::2Xnls-TagRFP::unc-54 3′ UTR* and *str-2p::myr-mCherry*. The AWC^ON^ cell was identified as *str-2p::myr-mCherry* positive and *odr-3p::2XTagRFP* positive (D′). The AWC^OFF^ cell was defined as *str-2p::myr-mCherry* negative and *odr-3p::2Xnls-TagRFP* positive (E′). (D″) Merge of D and D′ images from the same cell. (E″) Merge of E and E′ images from the same cell. (F) Quantification of normalized GFP expression in AWC^ON^ and AWC^OFF^ cells. Normalized GFP expression was determined by calibrating GFP intensity with 2Xnls-TagRFP intensity of the same cell. All constructs, except for *odr-3p::GFP::tir-1 3′ UTR*, contain the *unc-54* 3′ UTR. All images were taken from first stage larvae. The single focal plane with the brightest fluorescence in each AWC was selected from the acquired image stack and measured for fluorescence intensity. Each animal was categorized into one of three categories: AWC^ON^ = AWC^OFF^, AWC^ON^>AWC^OFF^, and AWC^OFF^>AWC^ON^ based on the comparison of GFP intensities between AWC^ON^ and AWC^OFF^ cells of the same animal. We did not observe any animals that fell into the “AWC^ON^ = AWC^OFF^” category from our GFP intensity analysis. Total number of animals for each category was tabulated and analyzed as described [86]. *p*-values were calculated using *X* ^2^ test. Error bars represent standard error of proportion. Arrows indicate the AWC cell bodies. Arrowheads represent myr-TagRFP or myr-mCherry signal. Scale bar, 2 µm.

We then investigated whether *mir-71*, when detected in both AWC neurons, has differential expression levels between AWC^ON^ and AWC^OFF^. Transgenic animals expressing *mir-71p::GFP*, *ceh-36p::myr-TagRFP* (myristoylated TagRFP marker of AWC^ON^ and AWC^OFF^), and *str-2p::2Xnls-TagRFP* (nucleus-localized TagRFP marker of AWC^ON^) were generated and analyzed in the L1 stage (Figure 4A, 4A′, 4A″, 4B, 4B′, and 4B″). The *ceh-36* promoter is expressed in AWCL, AWCR, ASEL, and ASER [50], [51]. *mir-71p::GFP* expression was significantly higher in the AWC^ON^ cell than in the AWC^OFF^ cell in 71% of the animals (*p*<0.001; Figure 4C). To confirm this result, we generated transgenic animals expressing *mir-71p::NZGFP*, *odr-3p::CZGFP*, and *str-2p::2Xnls-TagRFP* in which reconstituted GFP (recGFP) [52] expression from two split GFP polypeptides, NZGFP and CZGFP, was restricted mainly in the two AWC cells. Consistent with the *mir-71p::GFP* result, recGFP expression was significantly higher in the AWC^ON^ cell than in the AWC^OFF^ cell in 81% of the animals (*p*<0.001; Figure S4). Together, these results suggest that *mir-71* is expressed at a higher level in the AWC^ON^ than in the AWC^OFF^ cell. The higher expression of *mir-71* in the AWC^ON^ cell is consistent with the role of *mir-71* in promoting the AWC^ON^ fate.

### *tir-1* expression is downregulated through its 3′ UTR in the AWC^ON^ cell

The suppression of gene expression by *mir-71* through the *tir-1* 3′ UTR (Figure 2B and 2C) and the role of *mir-71* in promoting the AWC^ON^ fate (Figure 1C and 1E) suggest that gene expression through the *tir-1* 3′ UTR may be downregulated in the AWC^ON^ cell. To investigate this possibility, transgenic animals expressing *odr-3p::GFP::tir-1* 3′ *UTR* (GFP reporter of the *tir-1* 3′ UTR regulation in both AWCs), *odr-3p::2Xnls-TagRFP::unc-54 3′ UTR* (nucleus-localized TagRFP marker of both AWC^ON^ and AWC^OFF^), and *str-2p::myr-mCherry* (myristoylated mCherry marker of AWC^ON^) were generated and analyzed in the L1 stage (Figure 4D, 4D′, 4D″, 4E, 4E′, and 4E″). The GFP intensity was normalized to the nucleus-localized TagRFP intensity measured in the same AWC cell to account for variation in focal plane and promoter activity. Normalized GFP intensity was significantly lower in the AWC^ON^ cell than in the AWC^OFF^ cell in more than 85% of the animals (*p*<0.02; Figure 4F). These results suggest that the expression of *tir-1* is downregulated in the AWC^ON^ cell, consistent with a higher expression level of *mir-71* in AWC^ON^ and downregulation of *tir-1* expression by *mir-71*.

### *mir-71* acts cell-autonomously to promote the AWC^ON^ identity

To determine the site of *mir-71* action, mosaic animals in which the two AWC neurons have differential *mir-71* activity were used to ask whether *mir-71* acts in the future AWC^ON^ cell or the future AWC^OFF^ cell. Mosaic animals were generated by random and spontaneous mitotic loss of an unstable transgene expressing the *mir-71(OE)* construct *odr-3p::mir-71* and a mosaic marker *odr-1p::DsRed* that showed which AWC cells retained the transgene. We specifically looked for the mosaic animals in which only one of the two AWC neurons expressed the *mir-71(OE)* transgene; this cell was identified by expression of the DsRed marker.

**Figure 5:**
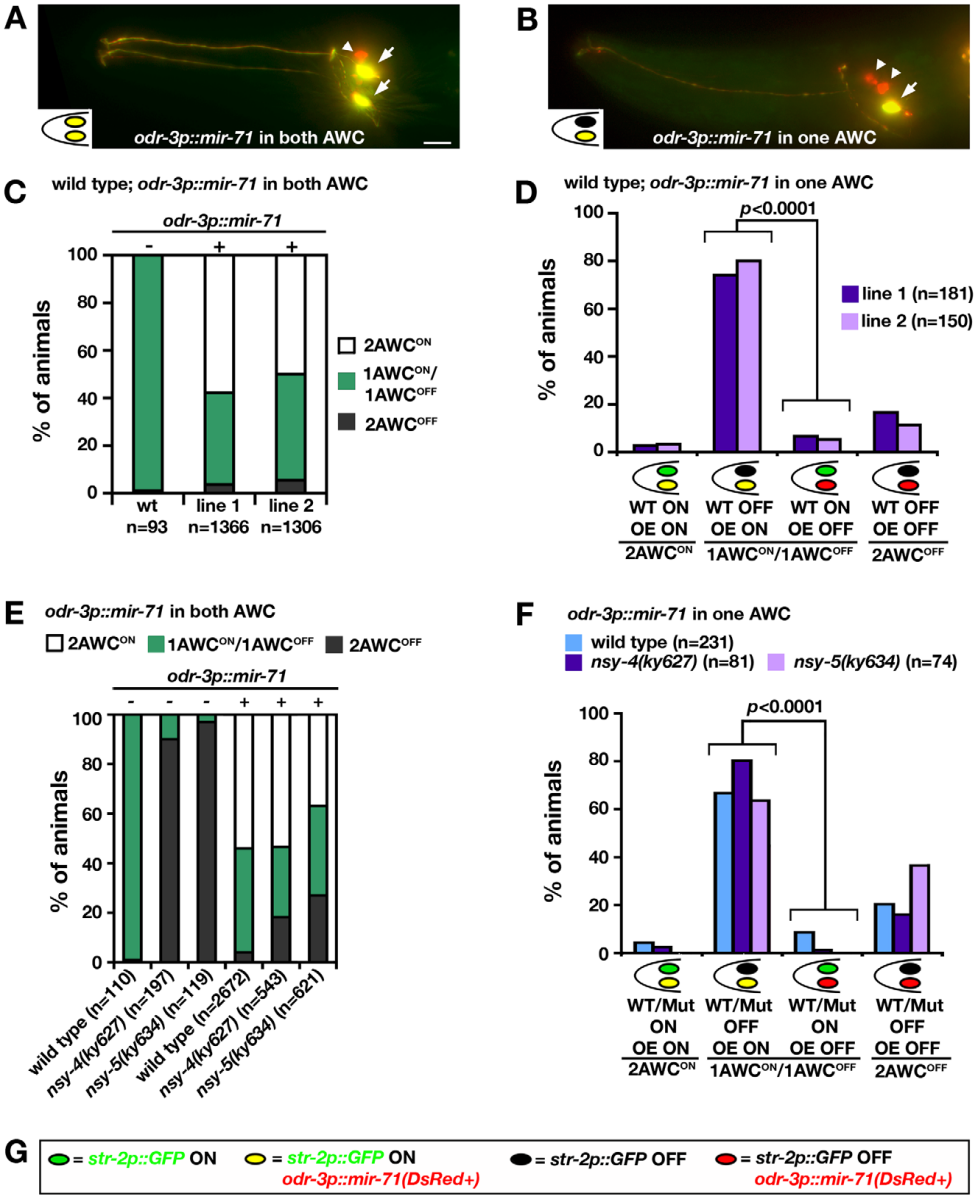
*mir-71* acts cell-autonomously to promote AWC^ON^. (A, B) Projections of wild-type animals expressing an integrated *str-2p::GFP* transgene (green) and an unstable transgenic array containing *odr-3p::mir-71* and *odr-1p::DsRed* (red). AWC neurons with co-expression of GFP and DsRed appear yellow. Arrows, AWC cell body; arrowheads, AWB cell body; scale bar, 10 µm. (C, E) AWC phenotypes of wild type (C), *nsy-4(ky627)*, and *nsy-5(ky634)* mutants (E) expressing the transgene *odr-3p::mir-71; odr-1p::DsRed* in both AWC neurons. + and − indicate the presence and absence of the transgene *odr-3p::mir-71*, respectively. (D, F) AWC phenotypes of wild-type (D) and mutant (F) mosaic animals expressing the transgene *odr-3p::mir-71; odr-1p::DsRed* in one AWC neuron. Two independent transgenic lines were analyzed in wild type, *nsy-4(ky627)*, and *nsy-5(ky634)* mutants in (C–F). Results from two independent lines were similar and thus were combined in (E, F). *Z*-test was used to calculate *p* values. (G) Color codes for AWC neurons in (A), (B), (D), and (F).

Mosaic analysis was first performed in transgenic lines expressing the *mir-71(OE)* transgene in a wild-type background. Expression of the *mir-71(OE)* transgene in both AWC neurons resulted in a 2AWC^ON^ phenotype (Figure 5A and 5C). When the *mir-71(OE)* transgene was retained in only one of the two AWC neurons, the *mir-71(OE)* AWC neuron became AWC^ON^ and wild-type AWC neuron became AWC^OFF^ in the majority of these mosaic animals (*p*<0.0001; Figure 5B and 5D). This result is consistent with a significant cell-autonomous requirement for *mir-71* in the AWC^ON^ cell to regulate its identity, which is opposite to the cell autonomous function of *tir-1* in regulation of the AWC^OFF^ identity. This result suggests that the AWC cell with higher *mir-71* activity can prevent the contralateral AWC cell from becoming AWC^ON^ and that *mir-71* may play a role in a negative-feedback signal sent from pre-AWC^ON^ to pre-AWC^OFF^. Similar results were obtained from previous mosaic analysis of *nsy-4* and *nsy-5* [18], [19].

NSY-4 claudin-like protein and NSY-5 gap junction protein are the two parallel signaling systems that antagonize the calcium signaling pathway to specify the AWC^ON^ identity [18], [19]. To determine whether *mir-71* acts downstream of *nsy-4* and *nsy-5* to promote AWC^ON^, mosaic analysis was performed with the *mir-71(OE)* transgene in *nsy-4(ky627)* and *nsy-5(ky634)* mutants. Loss-of-function mutations in *nsy-4* and *nsy-5* caused a 2AWC^OFF^ phenotype (Figure 5E) [18], [19], opposite to the *mir-71(OE)* 2AWC^ON^ phenotype. Overexpression of *mir-71* in both AWC neurons significantly suppressed the 2AWC^OFF^ phenotype of *nsy-4(ky627)* and *nsy-5(ky634)* mutants. In addition, *nsy-4(ky627); mir-71(OE)* and *nsy-5(ky634); mir-71(OE)* animals resembled the *mir-71(OE)* parent more closely than the *nsy-4(ky627)* or *nsy-5(ky634)* parent, but mixed phenotypes were observed (Figure 5E). These results suggest that *mir-71* mainly acts at a step downstream of *nsy-4* and *nsy-5* to promote AWC^ON^. In the majority of the mosaic animals retaining the *mir-71(OE)* transgene in only one of the two AWC neurons, the *mir-71(OE)* AWC neuron expressed *str-2p::GFP* and the other AWC neuron did not (Figure 5F). This significant cell-autonomous requirement for *mir-71* in the future AWC^ON^ neuron in *nsy-4(ky627)* and *nsy-5(ky634)* mutants is the same as in the wild-type background. These results suggest that *mir-71* acts cell autonomously downstream of *nsy-4* and *nsy-5* to promote the AWC^ON^ identity.

### The stability of mature *mir-71* is dependent on *nsy-4* and *nsy-5*

*alg-1* mutants had overaccumulation of premature *mir-71* and underaccumulation of mature *mir-71*, indicating that ALG-1/Argonaute-like protein is required for processing of *mir-71* from premature form into the mature form [53]. *alg-1(gk214)* single mutants had wild-type *str-2p::GFP* expression. However, *alg-1(gk214)* significantly suppressed the 2AWC^ON^ phenotype of *mir-71(OE)* and caused a weak 2AWC^OFF^ phenotype in *alg-1(gk214);mir-71(OE)* animals (*p*<0.001; Figure 1E). In addition, *alg-1(gk214)*, like *mir-71(n4115)* mutants, also significantly suppressed the 2AWC^ON^ phenotype of *tir-1(ky388ts)* mutants (*p*<0.05; Figure 1E). These results suggest that *alg-1* is required for *mir-71* function in the AWC^ON^ cell.

Consistent with previous northern blot analysis [53], we found a significantly reduced level of mature *mir-71* in *alg-1(gk214)* mutants (*p*<0.001; Figure S5A) using a stem-loop RT-PCR technique designed for specific quantification of mature miRNAs [54]. In addition, mature *mir-71* was not detected in *mir-71(n4115)* mutants (Figure S5B), suggesting that *mir-71(n4115)* is a null allele. Since *mir-71* is expressed broadly in the animal [48], [49] (Figure 3A), we introduced the AWC-expressing transgene *odr-3p::mir-71* in *mir-71(n4115)* mutants and used stem-loop RT-PCR to assay the level of mature *mir-71* mainly in AWC cells (Figure S5B).

To determine if the maturation and/or the stability of *mir-71* in AWCs is regulated by the signaling molecules that act upstream of *tir-1*, we assayed the level of mature *mir-71* in *mir-71(n4115); nsy-4(ky627)* double mutants, *mir-71(n4115); nsy-5(ky634)* double mutants, and *mir-71(n4115); unc-36(e251)* double mutants containing the AWC *mir-71(OE)* transgene using stem-loop RT-PCR (Text S1). The level of mature *mir-71* was significantly reduced in *nsy-4(ky627)* (*p* = 0.015) and *nsy-5(ky634)* (*p*<0.0001) mutants compared with control, but was not significantly different between control and *unc-36(e251)* mutants (Figure S5B). The decreased level of mature *mir-71* was not due to reduced transmission rates of the *odr-3p::mir-71* transgene (Figure S6A) or downregulation of the *odr-3* promoter in *nsy-4(ky627)* and *nsy-5(ky634)* mutants (Figure S6B). These results suggest that *nsy-4* and *nsy-5* are required for the generation and/or the stability of mature *mir-71*.

**Figure 6:**
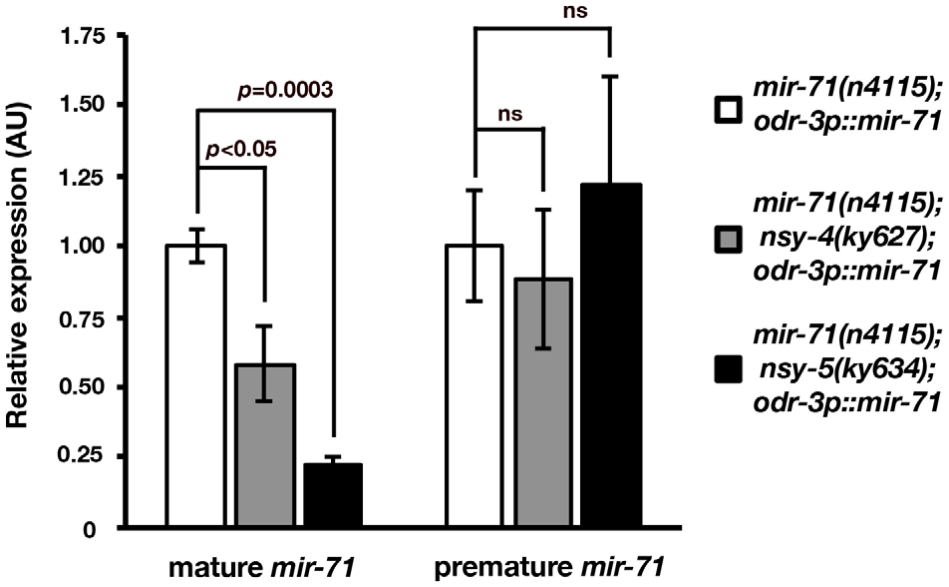
Mature *mir-71* level is decreased in *nsy-4* and *nsy-5* mutants. Stem-loop RT-qPCR analysis of mature and premature *mir-71* expression in *mir-71(n4115)*, *mir-71(n4115); nsy-5(ky634)*, and *mir-71(n4115); nsy-4(ky627)* mutants expressing the *odr-3p::mir-71* transgene in AWC. The expression levels of both premature and mature *mir-71* were normalized to those of the actin-related gene, *arx-1*. AU, arbitrary unit. Relative expression was set to one for *mir-71(n4115); odr3p::mir-71* and was normalized accordingly for other samples. *p* values were calculated using Student's *t*-test. ns, not significant (*p* = 0.6–0.7). Error bars represent standard error of the mean.

**Figure 7:**
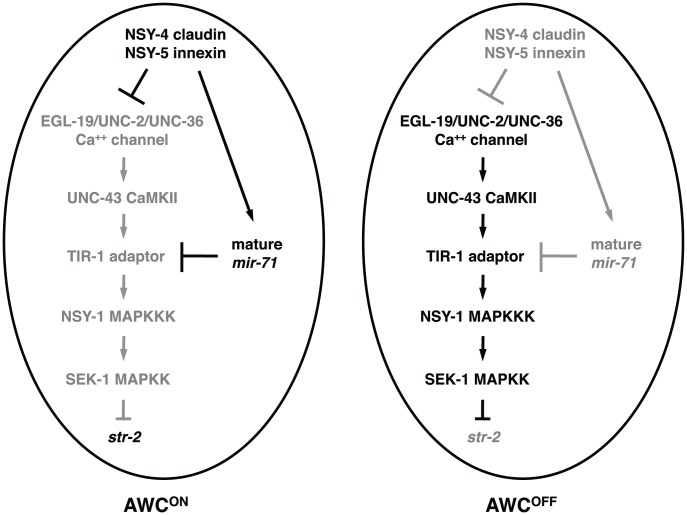
Model for *mir-71* function in AWC asymmetry. In the default AWC^OFF^ cell, *tir-1* acts in a calcium-regulated kinase signaling pathway to represses the expression of the AWC^ON^ marker *str-2*. Both *nsy-4* and *nsy-5* act to increase the level of mature *mir-71*, which results in downregulation of *tir-1* expression and subsequent de-repression of *str-2* gene expression in the cell that becomes AWC^ON^. Gray is used to indicate the gene product is less active or inactive.

To further determine whether *nsy-4* and *nsy-5* regulate the formation and/or the stability of mature *mir-71*, we performed stem-loop RT-qPCR to quantify the level of premature and mature *mir-71* in *mir-71(n4115)* mutants, *mir-71(n4115); nsy-4(ky627)* double mutants, and *mir-71(n4115); nsy-5(ky634)* double mutants containing the AWC *mir-71(OE)* transgene. Consistent with stem-loop RT-PCR results (Figure S5B), the abundance of mature *mir-71* was significantly decreased in *nsy-4(ky627)* (*p*<0.05) and *nsy-5(ky634)* (*p* = 0.0003) mutants (Figure 6). However, the level of premature *mir-71* was not significantly different between control and *nsy-4(ky627)* as well as *nsy-5(ky634)* mutants (Figure 6). These results suggest that the stability, but not the generation, of mature *mir-71* is reduced in *nsy-4(ky627)* and *nsy-5(ky634)* mutants, and are consistent with a model in which *nsy-4* and *nsy-5* promotes the stability of mature *mir-71* for downregulation of *tir-1* in the future AWC^ON^ cell (Figure 7).

*mir-71* is expressed at a higher level in the AWC^ON^ cell than in the AWC^OFF^ cell (Figure 4A–4C), suggesting that *mir-71* is differentially regulated at the transcriptional level in the two AWC cells. To determine if *nsy-4* and *nsy-5* also regulate differential expression levels of *mir-71* between the two AWC cells, we crossed the transgene (Figure 4A–4C) containing *mir-71p::GFP*, *ceh-36p::myr-TagRFP*, and *str-2p::2Xnls-TagRFP* into *nsy-4(ky627)* and *nsy-5(ky634)* mutants. Since the AWC^ON^ marker *str-2* is not expressed in *nsy-4(ky627)* or *nsy-5(ky634)* mutants, we analyzed and compared the expression levels of *mir-71p::GFP* between the two AWC cells in the mutants, instead of comparing the expression level between AWC^ON^ and AWC^OFF^ (Figure 4A–4C). We found that *mir-71* was also differentially expressed between the two AWC cells in *nsy-4(ky627)* and *nsy-5(ky634)* mutants (Figure S7), like in wild-type animals. These results suggest that differential regulation of *mir-71* transcription in the two AWC cells is not dependent on *nsy-4* or *nsy-5*.

## Discussion

Stochastic cell fate acquisition in the nervous system is a conserved but poorly understood phenomenon [1]. Here, we report that the miRNA *mir-71* is part of the pathway that controls stochastic left-right asymmetric differentiation of the *C. elegans* AWC olfactory neurons through downregulating the expression of *tir-1*, encoding the TIR-1/Sarm1 adaptor protein in a calcium signaling pathway. In addition, we have linked NSY-4/claudin- and NSY-5/innexin-dependent stability of mature *mir-71* to downregulation of calcium signaling in stochastic AWC neuronal asymmetry. Previous studies have identified the role of miRNAs in reproducible, lineage-based asymmetry of the *C. elegans* ASE taste neuron pair, in which the miRNA expression pattern is largely fixed along the left-right axis [8], [9], [55]. This study provides one of the first insights into miRNA function in stochastic left-right asymmetric neuronal differentiation, in which the miRNA expression pattern is not fixed and is likely regulated by the stochastic signaling event driving random asymmetry.

The seed match between *mir-71* and the *tir-1* 3′ UTR is conserved between *C. elegans* and *C. briggsae*. However, the *str-2* promoters share little sequence similarity between *C. elegans* and *C. briggsae*. The *C. elegans str-2* promoter GFP reporter, when expressed in *C. briggsae*, does not show detectable GFP expression in AWC neurons in embryos, first stage larvae, or adults (data not shown). This result suggests that the transcriptional regulation of *str-2* has diverged in *C. briggsae*.

*mir-71* has been implicated in various cell biological and developmental processes including promotion of longevity, resistance to heat and oxidative stress, DNA damage response, control of developmental timing, dauer formation, and recovery from dauer [47], [56]–[60]. However, it is largely unknown how *mir-71* functions to regulate these biological processes. RNA interference (RNAi) of *tir-1* did not affect *C. elegans* longevity [61], suggesting that *mir-71* may regulate distinct target genes for different functions.

miRNAs are important post-transcriptional and translational regulators of gene expression during development and disease. Several miRNA target prediction algorithms such as MicroCosm Targets, TargetScan, PicTar, and mirWIP provide useful tools with which to identify potential target genes of miRNAs [62]. However, many miRNAs have redundant functions and therefore give subtle or no phenotypes when mutated [37]–[40]. Overexpression approach or phenotypic analysis of miRNA mutants in sensitized genetic backgrounds have been successful in elucidating the role of miRNAs for which null mutants are not available or functional redundancy is a potential problem [5], [8], [38]–[40], [42], [63]–[65]. Using miRNA target prediction programs, we identified *mir-71* and five other miRNAs as potential regulators of the calcium-regulated UNC-43 (CaMKII)/TIR-1/NSY-1 (MAPKKK) signaling pathway. Through an overexpression approach and functional analysis of *mir-71(n4115)* mutants in sensitized genetic backgrounds, we revealed the role of *mir-71* in genetic control of the AWC^ON^ identity.

miRNAs that share the same sequence identity in their seed regions and could be potentially capable of downregulating the same set of target genes are grouped as members of a family [66]–[69]. Some miRNA family members have been shown to function redundantly and work together to regulate specific developmental processes [37], [38], [70]–[74]. However, many families of miRNAs did not show synthetic phenotypes, indicating that most miRNA families act redundantly with other miRNAs, miRNA families, or non-miRNA genes [38]. Since there is only one *mir-71* family member identified, the absence of an AWC phenotype in *mir-71(n4115)* single mutants suggests that *mir-71* may act redundantly with other miRNA family members or non-miRNA genes to regulate calcium signaling in AWC asymmetry. *dcr-1*, encoding the ribonuclease III enzyme Dicer, is required for processing of premature miRNAs to mature miRNAs [28]. *dcr-1(ok247)* null mutants had wild-type AWC asymmetry (data not shown). This result suggests that the *dcr-1* mutation may cause simultaneous knockdown of several miRNAs (including *mir-71*) with opposite functions in AWC asymmetry, thereby masking the role of *mir-71* and its redundant miRNAs in AWC asymmetry.

The UNC-76 axon guidance molecule and NSY-4 claudin-like protein act to antagonize the calcium-regulated signaling pathway to generate the AWC^ON^ identity [12], [19]. We found that *mir-71(n4115)* mutants significantly suppressed the 2AWC^ON^ phenotype of *nsy-4(OE)* and enhanced the 2AWC^OFF^ phenotype of *nsy-4(ky627)* and *unc-76(e911)* mutants. These results suggest an alternative mechanism for functional redundancy of *mir-71* in AWC asymmetry. *mir-71* may act in parallel with other regulatory pathways downstream of *unc-76* and *nsy-4* to downregulate the calcium signaling pathway in the AWC^ON^ cell. Functional redundancy of miRNAs and other regulatory pathways has been demonstrated by a previous study suggesting that *Drosophila* miR-7 may act in parallel with a protein-turnover mechanism to downregulate the transcriptional repressor Yan in the fly eye [42].

Our results suggest that *mir-71* is regulated at transcriptional and post-transcriptional levels in AWC. At the transcriptional level, *mir-71* is expressed at a higher level in the AWC^ON^ cell than in the AWC^OFF^ cell. This transcriptional bias of *mir-71* is not dependent on NSY-4 claudin-like protein or NSY-5 innexin gap junction protein. The mechanisms that regulate differential expression of *mir-71* in the two AWC cells are yet to be elucidated. At the post-transcriptional level, the stability of mature *mir-71* is dependent on *nsy-4* and *nsy-5*. It is possible that *nsy-4* and *nsy-5* may antagonize the miRNA turnover pathway to increase the level of mature *mir-71*. The *C. elegans* 5′→3′ exoribonuclease XRN-2 has been implicated in degradation of mature miRNAs released from Argonaute [75]. However, *xrn-2(RNAi)* animals did not show AWC phenotypes (data not shown), suggesting that the stability of mature *mir-71* may be independent of *xrn-2*.

The TIR-1/Sarm1 adaptor protein assembles a calcium-regulated signaling complex at synaptic regions to regulate the default AWC^OFF^ identity [16]. Downregulation of the TIR-1 adaptor protein by *mir-71* and other parallel pathways may represent an efficient mechanism to inhibit calcium signaling in the cell becoming AWC^ON^. Calcium signaling is one of the most common and conserved systems that control a wide range of processes including fertilization, embryonic pattern formation, cell proliferation, cell differentiation, learning and memory, and cell death during development and in adult life [76]. In addition, calcium signaling is implicated in left-right patterning in several tissues of different organisms [77]. It has been shown that negative regulation of calcium signaling by miRNAs is important for normal development and health [78]–[81]. In summary, our study and the studies from other labs demonstrate that downregulation of calcium signaling by miRNAs is one of the important mechanisms for cellular and developmental processes.

## Materials and Methods

### Strains

Wild-type strains were *C. elegans* variety Bristol, strain N2. Worm strains were generated and maintained by standard methods [82]. Mutations and integrated transgenes used are as follows: *kyIs140 [str-2p::GFP; lin-15(+)] I* [12], *kyIs323 [str-2p::GFP; ofm-1p::GFP] II* [22], *oyIs44 [odr-1p::DsRed] V* [51], *kyIs136 [str-2p::GFP; lin-15(+)] X* [12], *mir-71(n4115) I* [40], *nsy-5(ky634) I* [18], *ppfr-1(tm2180) unc-29(e1072) I* (gift from P. Mains, University of Calgary, Canada) [43], *rol-6(e187) II, tir-1(ky388ts) III* [16], *tir-1(ky648gf) III, tir-1(tm3036) III* [22], *unc-36(e251) III, dcr-1(ok247) III; nsy-4(ky616) IV, nsy-4(ky627) IV* [19], *unc-43(n498gf) IV, eri-1(mg366 IV), unc-76(e911) V, lin-15b(n744) X*, and *alg-1(gk214) X*.

Transgenes maintained as extrachromosomal arrays include *kyEx1127* [*odr-3p::nsy-4; myo-3p::DsRed*] [18], *vyEx149* [*odr-3p::mir-71* (25 ng/µl); *ofm-1p::DsRed* (20 ng/µl)], *vyEx187* [*mir-71p::YFP* (50 ng/µl); *elt-2p::CFP* (5 ng/µl)], *vyEx527, 528* [*odr-3p::mir-71* (50 ng/µl); *odr-1p::DsRed* (12 ng/µl); *ofm-1p::DsRed* (30 ng/µl)], *vyEx605, 606* [*odr-3p::GFP::tir-1 3′ UTR* (7.5 ng/µl); *elt-2p::CFP* (7.5 ng/µl)], *vyEx611, 615* [*odr-3p::GFP::unc-54 3′ UTR* (7.5 ng/µl); *elt-2p::CFP* (7.5 ng/µl)], *vyEx647* [*odr-3p::GFP::nsy-1 3′ UTR* (7.5 ng/µl); *elt-2p::CFP* (7.5 ng/µl)], *vyEx649, 651* [*odr-3p::GFP::tir-1 3′ UTRmut* (7.5 ng/µl); *elt-2p::CFP* (7.5 ng/µl)], *vyEx835, 836, 838* [*odr-3p::tir-1::tir-1 3′ UTRmut* (70 ng/µl); *elt-2p::CFP* (7.5 ng/µl)], *vyEx703, 720* [*odr-3p::tir-1::tir-1 3′ UTR* (70 ng/µl); *elt-2p::CFP* (7.5 ng/µl)], *vyEx905, 907* [*odr-3p::mir-74* (50 ng/µl); *ofm-1p::DsRed* (30 ng/µl)], *vyEx914, 917* [*odr-3p::mir-248* (50 ng/µl); *ofm-1p::DsRed* (30 ng/µl)], *vyEx915, 918* [*odr-3p::mir-72* (50 ng/µl); *ofm-1::DsRed* (30 ng/µl)], *vyEx916, 920, 921* [*odr-3p::mir-228* (50 ng/µl); *ofm-1p::DsRed* (30 ng/µl)], *vyEx922, 923, 924* [*odr-3p::mir-255* (50 ng/µl); *ofm-1::DsRed* (30 ng/µl)], *vyEx927, 931* [*mir-71p::GFP* (10 ng/µl); *ceh-36p::myr-TagRFP* (5 ng/µl); *str-2p::2Xnls-TagRFP* (25 ng/µl); *ofm-1p::DsRed* (30 ng/µl)], *vyEx1316, 1317* [*mir-71p::NZGFP* (30 ng/µl); *odr-3p::CZGFP* (15 ng/µl); *str-2p::2Xnls-TagRFP* (25 ng/µl); *ofm-1p::DsRed* (30 ng/µl)), *vyEx1318, 1319* [*nsy-5p::mir-248IR* (100 ng/µl); *odr-1p::DsRed* (15 ng/µl); *ofm-1p::DsRed* (30 ng/µl)], *vyEx1065* [*str-2p::myr-mCherry* (100 ng/µl); *ofm-1p::DsRed* (30 ng/µl)], *vyEx1097* [*odr-3p::2Xnls-TagRFP* (40 ng/µl); *pRF4(rol-6(su1006)* (50 ng/µl)], *vyEx1351, 1352* [*odr-3p::tir-1 5′ UTR::GFP::tir-1 3′ UTR* (15 ng/µl); *odr 3p::TagRFP::unc-54 3′UTR* (15 ng/µl); *elt-2p::CFP* (7.5 ng/µl)], and *vyEx1353, 1375* [*odr-3p::tir-1 5′UTR::GFP::tir-1 3′ UTRmut* (15 ng/µl); *odr 3p::TagRFP::unc-54 3′ UTR* (15 ng/µl); *elt-2p::CFP* (7.5 ng/µl)].

### Plasmid construction and germ line transformation

A 2476 bp PCR fragment of *mir-71* promoter was subcloned to make *mir-71p::YFP* and *mir-71p::GFP*. *mir-71p::NZGFP* was made by replacing GFP in *mir-71p::GFP* with a NZGFP fragment from TU#710 (Addgene) [52]. *odr-3p::CZGFP* was made by cloning a CZGFP fragment from TU#711 (Addgene) [52] into an *odr-3p* vector. *ceh-36p::myr-TagRFP*, in which the 1852 bp *ceh-36* promoter drives expression of myristoylated TagRFP, was generated by replacing *TagRFP* in *ceh-36p::TagRFP* [83] with *myr-TagRFP*. *odr-3p::2Xnls-TagRFP* was made by replacing the *str-2* promoter in *str-2p::2Xnls-TagRFP* [22] with the *odr-3* promoter [41]. *str-2p::myr-mCherry* was generated by replacing GFP in *str-2p::GFP* [12] with a *myr-mCherry* fragment. A 94 bp *mir-71* PCR fragment was subcloned to make *odr-3p::mir-71*. A 561 bp PCR fragment of the *tir-1* 3′ UTR, which represents the average length of the 3′ UTR in the majority of identified *tir-1* cDNA clones such as yk1473h08 (www.wormbase.org), was subcloned to make *odr-3p::tir-1::tir-1 3′ UTR* and *odr-3p::GFP::tir-1 3′ UTR*. miRNA target prediction algorithms including MicroCosm Targets, PicTar, and mirWIP use 300–590 bp of *tir-1* 3′ UTR for analysis. The predicted *mir-71* binding site, TCTTTC, in the *tir-1* 3′ UTR was mutated into CAGGCA using QuikChange II XL Site-Directed Mutagenesis Kit (Stratagene) to make *odr-3p::GFP::tir-1 3′ UTRmut*. *tir-1a* splice form was used for all *tir-1* constructs. *odr-3p::tir-1 5′ UTR::GFP::tir-1 3′ UTR* was made by cloning a 150 bp PCR fragment of *tir-1* 5′ UTR, amplified from wild-type embryo cDNA, into the *odr-3p::GFP::tir-1 3′ UTR*. *odr-3p::tir-1 5′ UTR::GFP::tir-1 3′ UTRmut* was made by replacing *GFP::tir-1 3′ UTR* in the *odr-3p::tir-1 5′ UTR::GFP::tir-1 3′ UTR* with *GFP::tir-1 3′ UTRmut*. To make shRNA anti-*mir-248* (*mir-248IR*), the sense and antisense oligos, each consisting of *mir-248* sense (24 nt) and antisense (24 nt) sequences that flank a 12 nt linker (loop) sequence, were designed (SBI System Biosciences) and annealed (IDT) as described. This hairpin construct was subcloned to make *nsy-5p::mir-248IR*. To generate transgenic strains, DNA constructs were injected into the syncytial gonad of adult worms as previously described [84].

### Quantification of fluorescence intensity

Z-stack images of transgenic animals expressing fluorescent markers were acquired using a Zeiss Axio Imager Z1 microscope equipped with a motorized focus drive and a Zeiss AxioCam MRm CCD digital camera. All animals of each set of experiments had the same exposure time for comparison of fluorescence intensity. The single focal plane with the brightest fluorescence in each AWC cell was selected from the acquired image stack and measured for fluorescence intensity. To measure fluorescence intensity, the outline spline tool in the Zeiss AxioVision Rel 4.7 image analysis software was used to draw around the AWC cell body (Figure 2B; Figure 4A, 4B, 4D, 4E; Figure S4A, S4B; and Figure S6B) or nucleus (Figure 2B, Figure 4D′ and 4E′) from captured images. To measure fluorescence intensity in dim GFP-expressing cells (Figure 4B and Figure S4B), the display contrast and brightness were adjusted to visualize and outline the cells. For each category of animals, images from a minimum of 10 animals were collected and analyzed.

### Genetic mosaic analysis

Mosaic analysis was performed as previously described [13], [18], [19], [25]. Transgenic lines expressing the *odr-3p::mir-71; odr-1p::DsRed* transgene were passed for minimum of six generations before scoring for mosaic animals. The same transgenic lines were crossed into *nsy-4(ky627)* and *nsy-5(ky634)* mutants for the analysis.

### qPCR for determining the relative transgene copy number

Three adult hermaphrodites from each *tir-1(OE)* transgene line were collected in 25 µl of worm lysis buffer (50 mM KCl, 0.01% gelatin, 10 mM Tris-HCl pH 8.3, 0.45% Tween 20, 0.45% NP-40, 2.5 mM MgCl_2_, 100 µg/ml Proteinase K). Collected worms were then incubated at −80°C for minimum of one hour, 65°C for one hour, and 95°C for 15 minutes. 5 µl of the worm lysate was used for subsequent qPCR with Fast SYBR Green Master Mix (Invitrogen). qPCR reactions were run in triplicate at 95°C for 3 minutes, followed by 45 cycles of 95°C for 30 seconds, 57°C for 30 seconds, and 72°C for 30 seconds on the CFX96 Real-Time PCR Detection System (Bio-Rad). PCR product was scanned for fluorescent signal at the end of each cycle and the C(T) values were obtained using the CFX Manager Software (Bio-Rad). The relative *tir-1(OE)* transgene copy number was determined using the 2[−Delta Delta C(T)] method as previously described [85] with the actin-related gene, *arx-1*, as internal control.

### Stem-loop RT–qPCR of premature and mature *mir-71*

Stem-loop RT-qPCR was performed as described [54] to detect and quantify relative expression levels of premature and mature *mir-71*. The *odr-3p::mir-71* transgenes used in genetic mosaic analysis were crossed into various genetic backgrounds. Total RNA samples were isolated from first stage larvae using RNeasy Mini kit (QIAGEN). Reverse transcription (RT) reactions were performed with 1 µg of total RNA, SuperScript III reverse transcriptase (Invitrogen), and RT primer (oligo d(T)_18_, premature *mir-71* stem-loop RT primer, or mature *mir-71* stem-loop RT primer). 1 µl of 1∶35 diluted reverse transcription product was used as template for subsequent qPCR reactions with Fast SYBR Green Master Mix (Invitrogen). All PCR reactions were run in triplicate at 95°C for 3 minutes, followed by 45 cycles of 95°C for 30 seconds, 51°C for 30 seconds, and 72°C for 30 seconds on the CFX96 Real-Time PCR Detection System (Bio-Rad). PCR product was scanned for fluorescent signal at the end of each cycle and the C(T) values were obtained using the CFX Manager Software (Bio-Rad). The actin-related gene, *arx-1*, was used as internal control to normalize variation between samples. Relative expression of premature and mature *mir-71* was analyzed using the 2[−Delta Delta C(T)] method as previously described [85]. Relative expression was set to one for *mir-71(n4115); odr3p::mir-71* and was normalized accordingly for other samples. Student's *t*-test was used to calculate statistical significance.

## Supporting Information

Figure S1 miRNAs predicted to target genes in the AWC calcium-mediated signaling pathway. (A) A list of miRNAs and target genes identified by four miRNA target prediction programs. Only the prediction that fits the two indicated criteria is listed. (B) AWC phenotypes caused by overexpression of candidate miRNAs listed in (A).
(TIF)

Figure S2 The effect of *mir-71* on GFP sensor constructs with the *tir-1* 3′ UTR or the *nsy-1* 3′ UTR. (A) Normalized GFP intensity in wild type and *mir-71(n4115)* mutants carrying the transgene of GFP sensor constructs with the *tir-1* 3′ UTR or the *unc-54* 3′ UTR (as negative control). (B) Normalized GFP intensity in wild type and *mir-71(OE)* animals expressing the transgene of a GFP sensor construct with the *nsy-1* 3′ UTR.
(TIF)

Figure S3 The *tir-1* 5′ UTR does not affect *mir-71(OE)*-mediated downregulation of gene expression through the *tir-1* 3′ UTR. (A, B) The average normalized GFP intensity in the AWC cell body of sensor constructs, driven by the *odr-3* promoter and the *tir-1* 5′ UTR, with the *tir-1* 3′ UTR (A) or the *tir-1* 3′ UTR mutated in the predicted *mir-71* target site (B), in wild type and *mir-71(OE)* animals. The GFP intensity of an individual cell was normalized to the TagRFP intensity of the internal control transgene *odr-3p::2Xnls-TagRFP::unc-54 3′ UTR* in the same cell in the first larval stage. For each sensor construct, the normalized GFP intensity in wild type was set as 1 arbitrary unit (AU) and the normalized GFP intensity in *mir-71(OE)* was calibrated to that in wild type. Two independent lines were analyzed for each sensor construct. Student's *t*-test was used for statistical analysis. Error bars, standard error of the mean. ns, not significant.
(TIF)

Figure S4 The expression level of *mir-71* is higher in the AWC^ON^ cell than in the AWC^OFF^ cell. (A, B) Images of recGFP expressed from *mir-71p::NZGFP* and *odr-3p::CZGFP*. (A′, B′) Images of *str-2p::2Xnls-TagRFP*. AWC^ON^ was identified as *str-2p::2Xnls-TagRFP* positive (A′). AWC^OFF^ was identified as *str-2p::2Xnls-TagRFP* negative (B′). (A″) Merge of A and A′ images from the same cell. (B″) Merge of B and B′ images from the same cell. (C) Quantification of recGFP expression in AWC^ON^ and AWC^OFF^ cells. All images were taken from first stage larvae. The single focal plane with the brightest fluorescence in each AWC was selected from the acquired image stack and measured for fluorescence intensity. Each animal was categorized into one of three categories: AWC^ON^ = AWC^OFF^, AWC^ON^>AWC^OFF^, and AWC^OFF^>AWC^ON^ based on the comparison of recGFP intensities between AWC^ON^ and AWC^OFF^ cells of the same animal. We did not observe any animals that fell into the “AWC^ON^ = AWC^OFF^” category from our recGFP intensity analysis. Total number of animals for each category was tabulated and analyzed as described [86]. *p*-values were calculated using *X* ^2^ test. Error bars represent standard error of proportion. Scale bar, 2 µm.
(TIF)

Figure S5 Stem-loop RT-PCR analysis of mature *mir-71* levels. (A, B) Representative images of stem-loop RT-PCR product of total RNA samples from adult worms (A) or enriched first stage larvae (B) in different genetic backgrounds. + and − indicate the presence and absence of the transgene *odr-3p::mir-71*, respectively. The actin-related gene *arx-1* was used as internal control to normalize the abundance of mature *mir-71*. All PCR reactions were run in triplicate. *p* values were calculated using Student's *t*-test. ns, not significant. Error bars represent standard error of the mean.
(TIF)

Figure S6 Control experiments to demonstrate that a decreased level of mature *mir-71* in *nsy-4(ky627)* and *nsy-5(ky634)* mutants is not caused by a reduced transmission rate of the *odr-3p::mir-71* extrachromosomal array or reduced activity of the *odr-3* promoter. (A) Transmission rates of the *odr-3p::mir-71* extrachromosomal array in *mir-71(n4115)*, *mir-71(n4115);nsy-4(ky627)*, and *mir-71(n4115);nsy-5(ky634)* mutants. Error bars represent the standard error of proportion. (B) Top: Representative images of *odr-3p::GFP* expression in AWC neurons of wild type, *nsy-4(ky627)*, and *nsy-5(ky634)* mutants at the first larval stage. Bottom: The average intensity of GFP in AWC neurons. Results from two independent *odr-3p::GFP* transgenic lines are shown. Error bars represent standard error of the mean. Scale bar, 10 µm.
(TIF)

Figure S7 Differential expression of *mir-71* in the two AWC cells is not dependent on *nsy-4* or *nsy-5*. The GFP intensity of *mir-71p::GFP* was compared between the two AWC cells of the same animal in wild-type, *nsy-4(ky627)*, and *nsy-5(ky634)* mutants. The percentage difference of *mir-71p::GFP* expression between the two AWC cells was determined by dividing the higher GFP intensity with the lower GFP intensity. Error bars represent the standard error of proportion.
(TIF)

Text S1 Supplemental Methods: Quantification of mature *mir-71* by stem-loop RT–PCR.
(DOCX)
