# Supplementary figures and images for: The MicroRNA mir-71 Inhibits Calcium Signaling by Targeting the TIR-1/Sarm1 Adaptor Protein to Control Stochastic L/R Neuronal Asymmetry in C. elegans
Source: PLoS Genet. 2012 Aug 2;8(8):e1002864. doi: 10.1371/journal.pgen.1002864 (PMC3410857; doi:10.1371/journal.pgen.1002864)

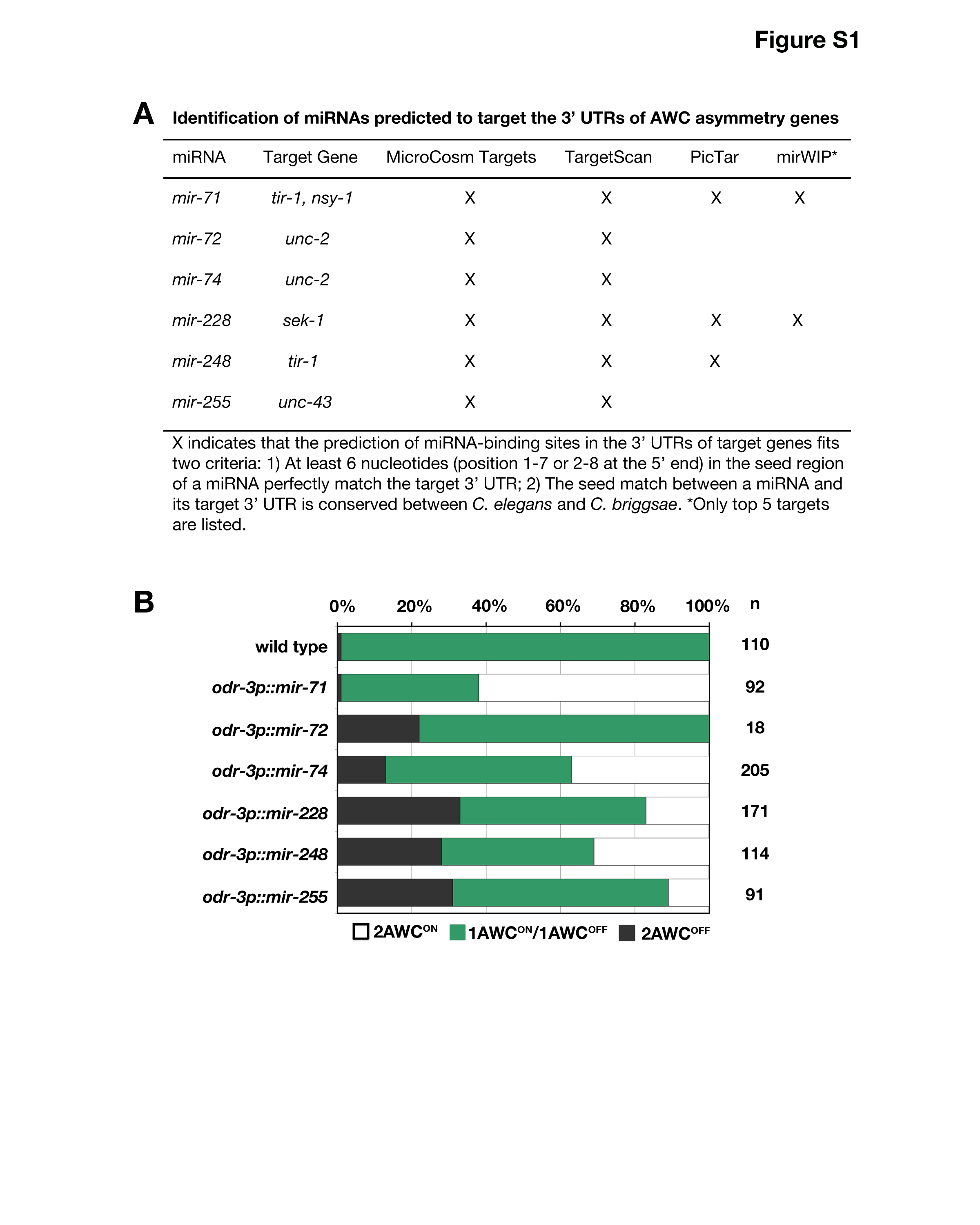

Supplement: Figure S1 — miRNAs predicted to target genes in the AWC calcium-mediated signaling pathway. (A) A list of miRNAs and target genes identified by four miRNA target prediction programs. Only the prediction that fits the two indicated criteria is listed. (B) AWC phenotypes caused by overexpression of candidate miRNAs listed in (A). (TIF) [file pgen.1002864.s001.tif]

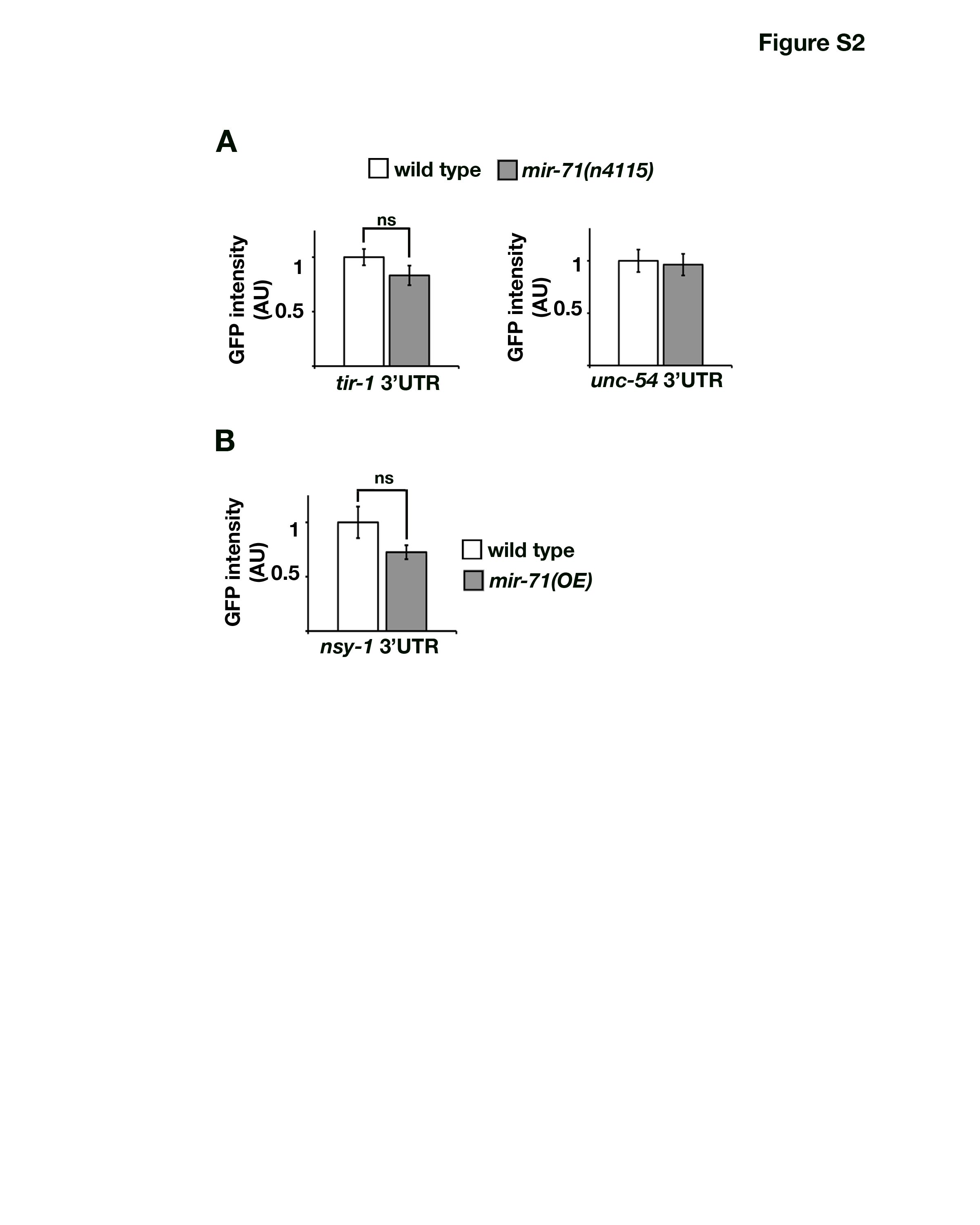

Supplement: Figure S2 — The effect of mir-71 on GFP sensor constructs with the tir-1 3′ UTR or the nsy-1 3′ UTR. (A) Normalized GFP intensity in wild type and mir-71(n4115) mutants carrying the transgene of GFP sensor constructs with the tir-1 3′ UTR or the unc-54 3′ UTR (as negative control). (B) Normalized GFP intensity in wild type and mir-71(OE) animals expressing the transgene of a GFP sensor construct with the nsy-1 3′ UTR. (TIF) [file pgen.1002864.s002.tif]

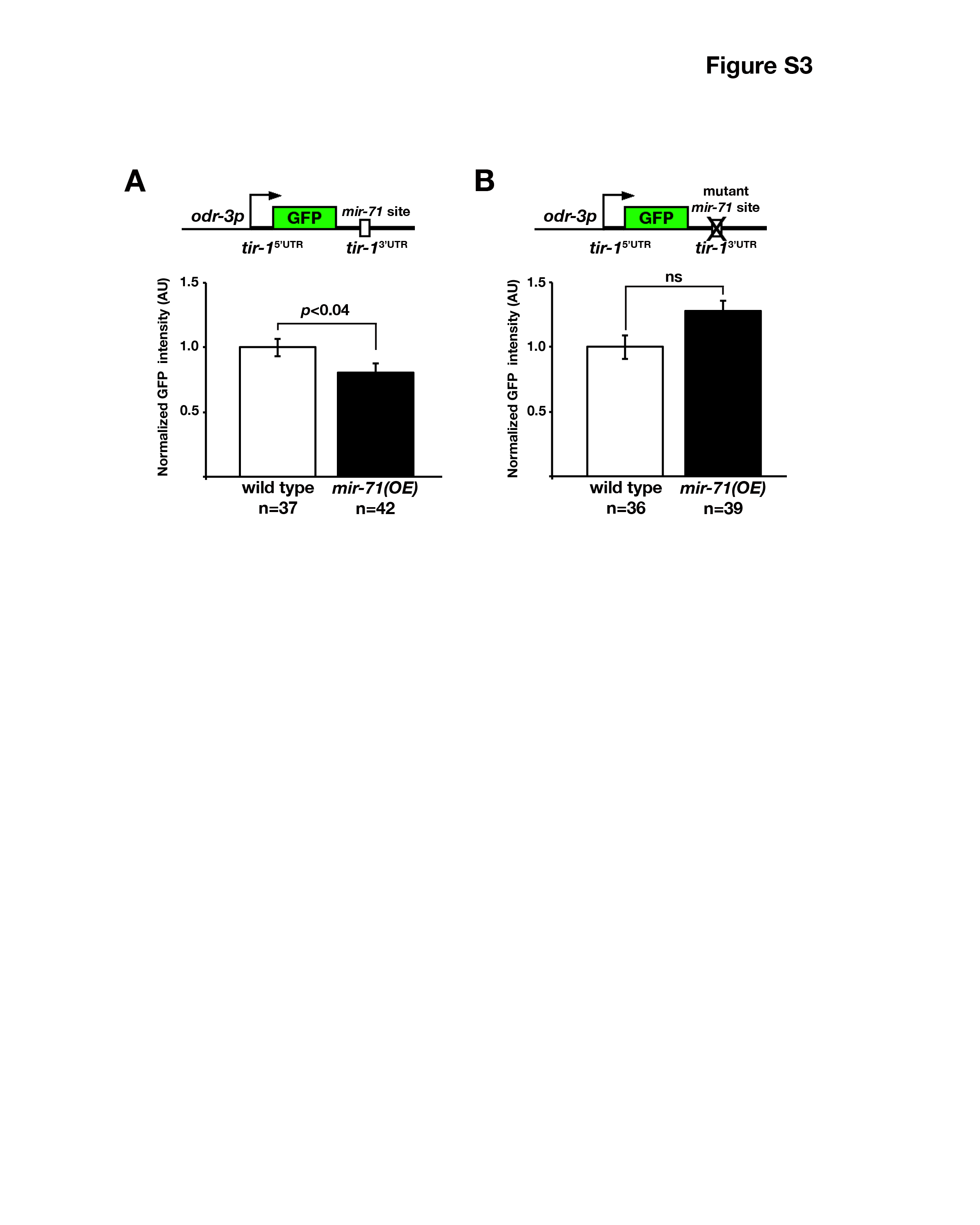

Supplement: Figure S3 — The tir-1 5′ UTR does not affect mir-71(OE)-mediated downregulation of gene expression through the tir-1 3′ UTR. (A, B) The average normalized GFP intensity in the AWC cell body of sensor constructs, driven by the odr-3 promoter and the tir-1 5′ UTR, with the tir-1 3′ UTR (A) or the tir-1 3′ UTR mutated in the predicted mir-71 target site (B), in wild type and mir-71(OE) animals. The GFP intensity of an individual cell was normalized to the TagRFP intensity of the internal control transgene odr-3p::2Xnls-TagRFP::unc-54 3′ UTR in the same cell in the first larval stage. For each sensor construct, the normalized GFP intensity in wild type was set as 1 arbitrary unit (AU) and the normalized GFP intensity in mir-71(OE) was calibrated to that in wild type. Two independent lines were analyzed for each sensor construct. Student's t-test was used for statistical analysis. Error bars, standard error of the mean. ns, not significant. (TIF) [file pgen.1002864.s003.tif]

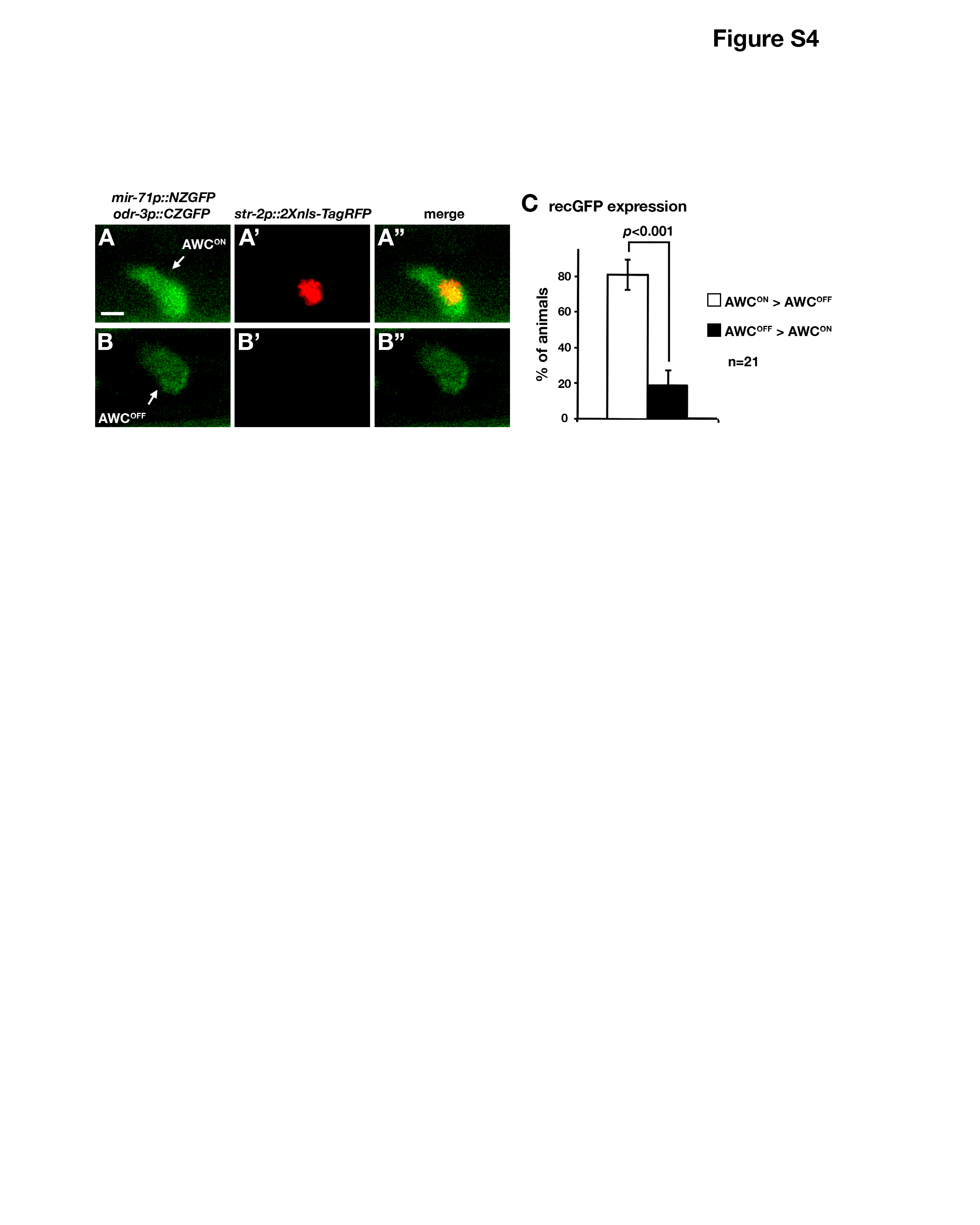

Supplement: Figure S4 — The expression level of mir-71 is higher in the AWCON cell than in the AWCOFF cell. (A, B) Images of recGFP expressed from mir-71p::NZGFP and odr-3p::CZGFP. (A′, B′) Images of str-2p::2Xnls-TagRFP. AWCON was identified as str-2p::2Xnls-TagRFP positive (A′). AWCOFF was identified as str-2p::2Xnls-TagRFP negative (B′). (A″) Merge of A and A′ images from the same cell. (B″) Merge of B and B′ images from the same cell. (C) Quantification of recGFP expression in AWCON and AWCOFF cells. All images were taken from first stage larvae. The single focal plane with the brightest fluorescence in each AWC was selected from the acquired image stack and measured for fluorescence intensity. Each animal was categorized into one of three categories: AWCON = AWCOFF, AWCON>AWCOFF, and AWCOFF>AWCON based on the comparison of recGFP intensities between AWCON and AWCOFF cells of the same animal. We did not observe any animals that fell into the “AWCON = AWCOFF” category from our recGFP intensity analysis. Total number of animals for each category was tabulated and analyzed as described [86]. p-values were calculated using X 2 test. Error bars represent standard error of proportion. Scale bar, 2 µm. (TIF) [file pgen.1002864.s004.tif]

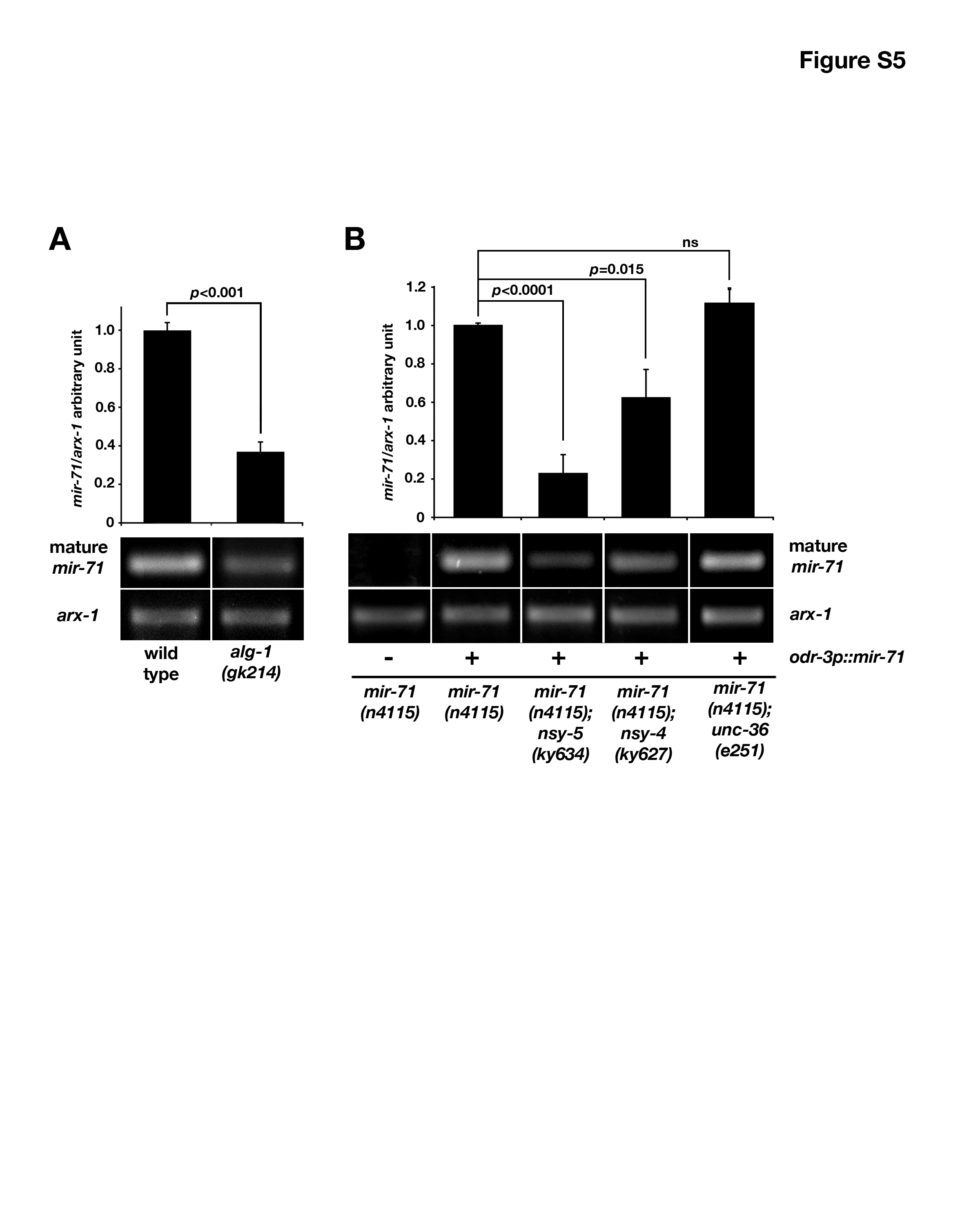

Supplement: Figure S5 — Stem-loop RT-PCR analysis of mature mir-71 levels. (A, B) Representative images of stem-loop RT-PCR product of total RNA samples from adult worms (A) or enriched first stage larvae (B) in different genetic backgrounds. + and − indicate the presence and absence of the transgene odr-3p::mir-71, respectively. The actin-related gene arx-1 was used as internal control to normalize the abundance of mature mir-71. All PCR reactions were run in triplicate. p values were calculated using Student's t-test. ns, not significant. Error bars represent standard error of the mean. (TIF) [file pgen.1002864.s005.tif]

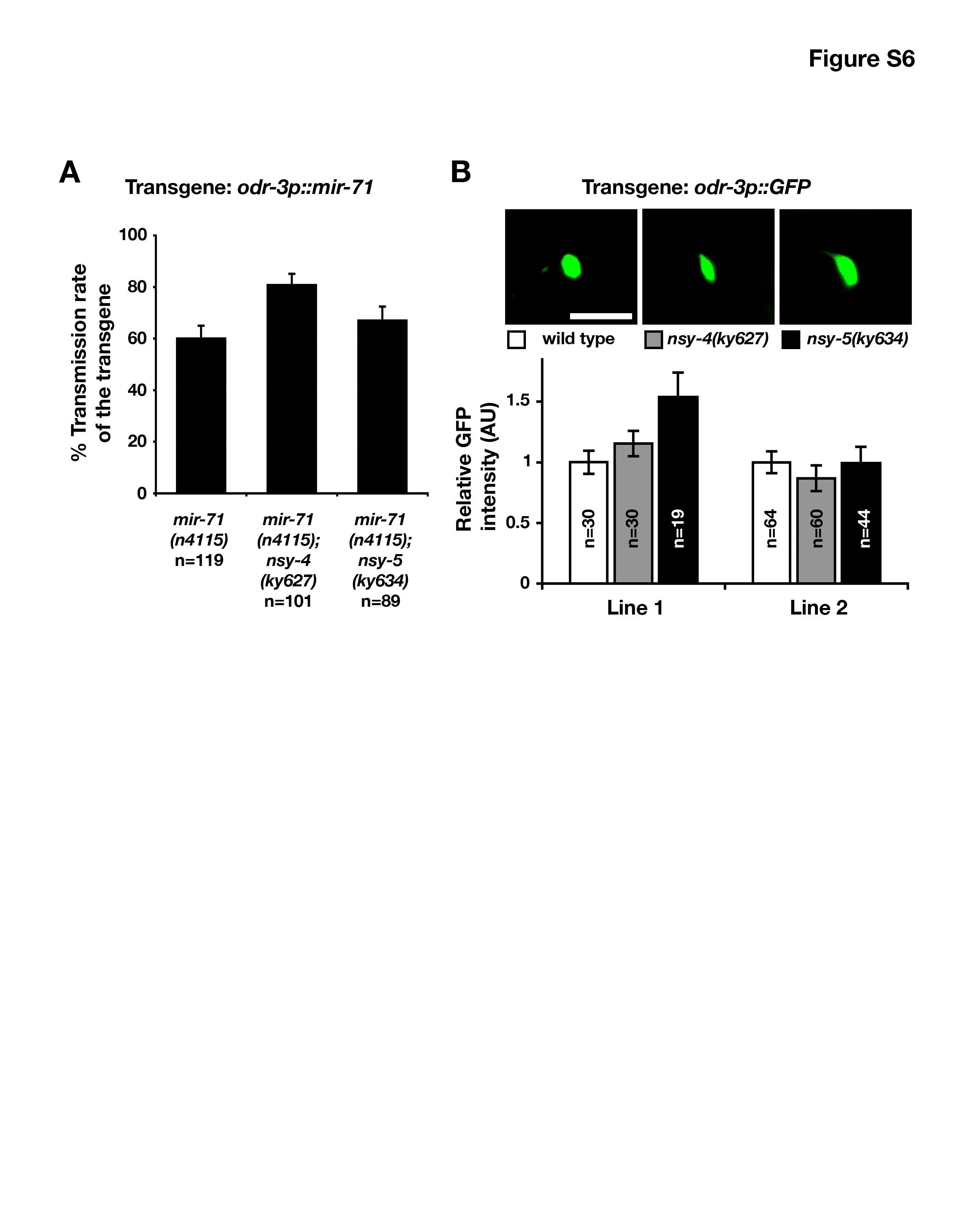

Supplement: Figure S6 — Control experiments to demonstrate that a decreased level of mature mir-71 in nsy-4(ky627) and nsy-5(ky634) mutants is not caused by a reduced transmission rate of the odr-3p::mir-71 extrachromosomal array or reduced activity of the odr-3 promoter. (A) Transmission rates of the odr-3p::mir-71 extrachromosomal array in mir-71(n4115), mir-71(n4115);nsy-4(ky627), and mir-71(n4115);nsy-5(ky634) mutants. Error bars represent the standard error of proportion. (B) Top: Representative images of odr-3p::GFP expression in AWC neurons of wild type, nsy-4(ky627), and nsy-5(ky634) mutants at the first larval stage. Bottom: The average intensity of GFP in AWC neurons. Results from two independent odr-3p::GFP transgenic lines are shown. Error bars represent standard error of the mean. Scale bar, 10 µm. (TIF) [file pgen.1002864.s006.tif]

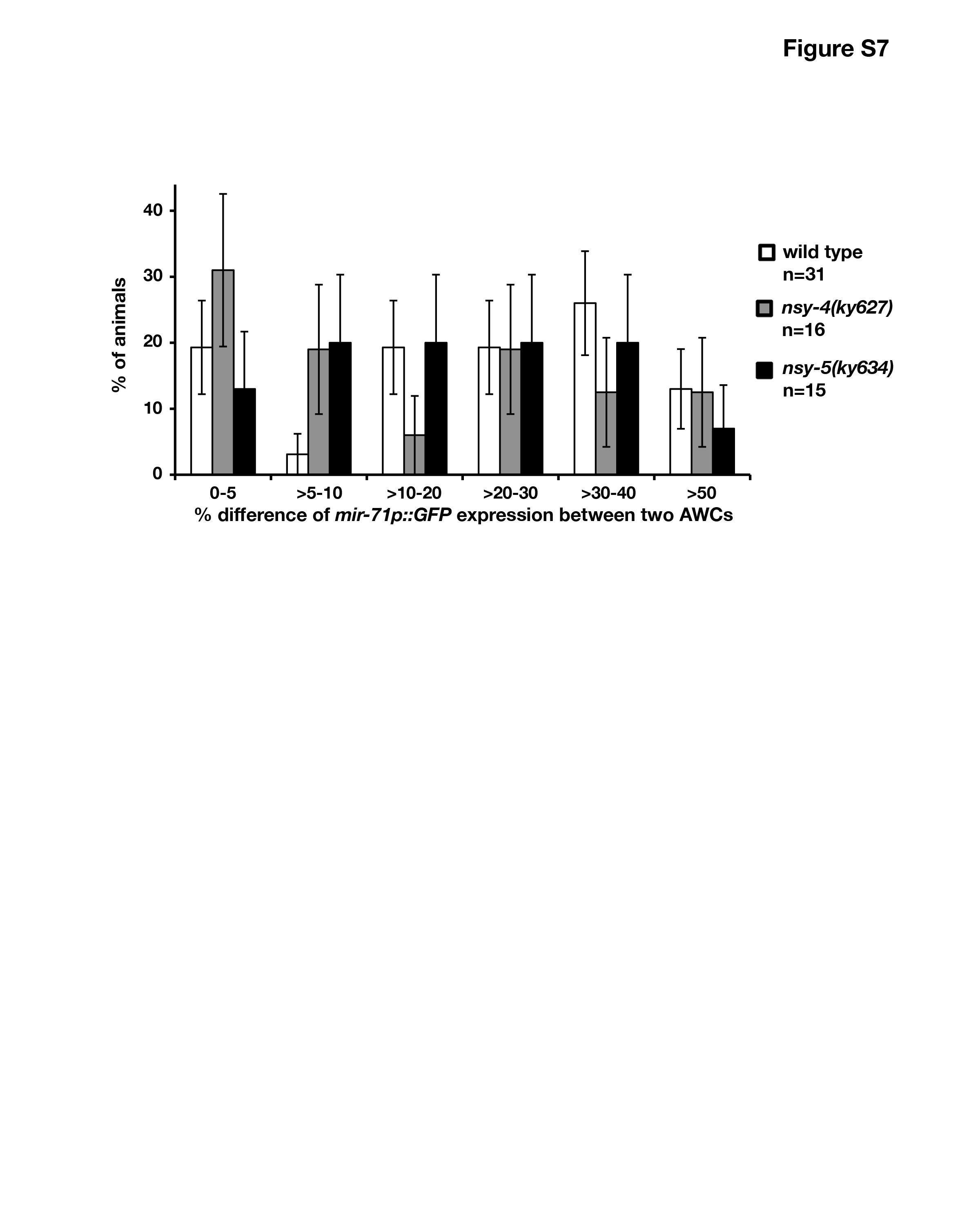

Supplement: Figure S7 — Differential expression of mir-71 in the two AWC cells is not dependent on nsy-4 or nsy-5. The GFP intensity of mir-71p::GFP was compared between the two AWC cells of the same animal in wild-type, nsy-4(ky627), and nsy-5(ky634) mutants. The percentage difference of mir-71p::GFP expression between the two AWC cells was determined by dividing the higher GFP intensity with the lower GFP intensity. Error bars represent the standard error of proportion. (TIF) [file pgen.1002864.s007.tif]
