# Supplementary material for: The MicroRNA mir-71 Inhibits Calcium Signaling by Targeting the TIR-1/Sarm1 Adaptor Protein to Control Stochastic L/R Neuronal Asymmetry in C. elegans
Source: PLoS Genet. 2012 Aug 2;8(8):e1002864. doi: 10.1371/journal.pgen.1002864 (PMC3410857; doi:10.1371/journal.pgen.1002864)
Supplement: Text S1 — Supplemental Methods: Quantification of mature mir-71 by stem-loop RT–PCR. (DOCX) [file pgen.1002864.s008.docx]

**Supplemental Methods**

**Quantification of mature *mir-71* by stem-loop RT-PCR**

Stem-loop RT was performed as described [[54](#_ENREF_54)] to detect and quantify the relative level of mature *mir-71*. Total RNA samples were isolated from adults (for Figure S5A experiments) or first stage larvae (for Figure S5B experiments) using QIAGEN RNeasy Mini kit. Reverse transcription (RT) reactions were performed with 1 μg of total RNA, SuperScript III reverse transcriptase (Invitrogen), and RT primer (oligo d(T)_18_ or *mir-71* stem-loop RT primer). 1-2 μl of the reverse transcription product was used as template for subsequent PCR reactions with iTaq DNA polymerase (Bio-Rad). All PCR reactions were run in triplicate at 95°C for 3 minutes, followed by 19-20 cycles (*mir-71*) or 22-25 cycles (*arx-1*) of 95°C for 30 seconds, 51°C for 30 seconds, and 72°C for 30 seconds. The cycle numbers were in the linear range of amplification. A 55 bp PCR fragment of mature *mir-71* was amplified using the *mir-71* RT product as template. The RT product generated from oligo d(T)_18_ was used to amplify a 122 bp fragment of the actin-related gene *arx-1,* which was used as internal control to normalize variation between samples. PCR product was resolved on a 3% agarose gel with ethidium bromide and imaged using Gel Logic Imaging System (Kodak). The intensity of the PCR product in the gel images was analyzed using the NIH ImageJ software. Student’s *t*-test was used to calculate statistical significance.
